# Galeon: A Biologically Active Molecule with In Silico Metabolite Prediction, In Vitro Metabolic Profiling in Rat Liver Microsomes, and In Silico Binding Mechanisms with CYP450 Isoforms

**DOI:** 10.3390/molecules25245903

**Published:** 2020-12-13

**Authors:** A. F. M. Motiur Rahman, Wencui Yin, Adnan A. Kadi, Yurngdong Jahng

**Affiliations:** 1 Department of Pharmaceutical Chemistry, College of Pharmacy, King Saud University, Riyadh 11451, Saudi Arabia; wyin@ksu.edu.sa (W.Y.); akadi@ksu.edu.sa (A.A.K.); 2 College of Pharmacy, Yeungnam University, Gyeongsan 38541, Korea; ydjahng@ynu.ac.kr

**Keywords:** galeon, cyclic-diarylheptanoid, galeon conjugates, galeon metabolites, galeon-CYP450 interactions

## Abstract

Galeon, a natural cyclic-diarylheptanoid (CDH), which was first isolated from *Myrica gale* L., is known to have potent cytotoxicity against A549 cell lines, anti-tubercular activity against *Mycobacterium tuberculosis* H37Rv, chemo-preventive potential, and moderate topoisomerase inhibitory activity. Here, in silico metabolism and toxicity prediction of galeon by CYP450, in vitro metabolic profiling study in rat liver microsomes (RLMs), and molecular interactions of galeon-CYP450 isoforms were performed. An in silico metabolic prediction study showed demethyl and mono-hydroxy galeon were the metabolites with the highest predictability. Among the predicted metabolites, mono-hydroxy galeon was found to have plausible toxicities such as skin sensitization, thyroid toxicity, chromosome damage, and carcinogenicity. An in vitro metabolism study of galeon, incubated in RLMs, revealed eighteen Phase-I metabolites, nine methoxylamine, and three glutathione conjugates. Identification of possible metabolites and confirmation of their structures were carried out using ion-trap tandem mass spectrometry. In silico docking analysis of galeon demonstrated significant interactions with active site residues of almost all CYP450 isoforms.

## 1. Introduction

**Figure 1:**
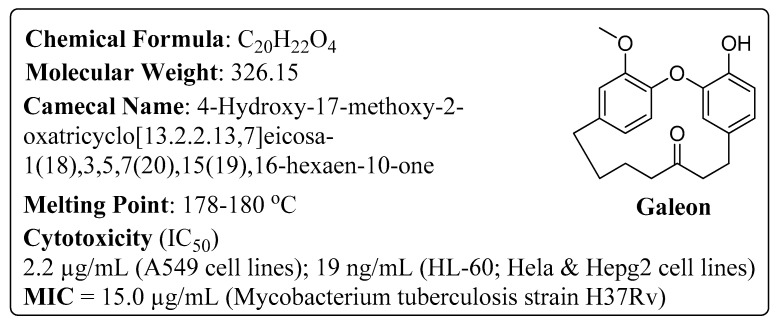
Chemical structure and properties of galeon.

Metabolic profiling of known/unknown and natural/synthetic molecules has become attractive to scientists due to the fact of its importance in drug discovery and development. Understanding how a compound is metabolized enables identification and analysis of its potential metabolites and guides the design of molecules with a reduced risk of drug-drug interactions or variations in exposure across the patient population. Drug metabolism can generate chemically reactive metabolites which are, in most cases, responsible for the unexpected toxicities of a drug, as they tend to react with cellular components [1,2], such as DNA and proteins, leading to mutagenicity, teratogenicity or carcinogenicity [3]. This is a major issue for patient safety and sometimes leads to drug withdrawals or use restriction. Therefore, it is important to study the metabolic profile before a drug molecule goes into clinical use [1,2]. In addition, bioavailability, which is partially attributed to the first-pass metabolism of a drug, plays an important role in ensuring a drug reaches its site of action to elicit its function. Based on the metabolic performance of a potential drug molecule, the clinical application can then proceed accordingly. Although a large number of natural/synthetic molecules have been reported to have potent biological activities, their further development is hampered due to the fact of their poor bioavailability and toxicity (i.e., generation of toxic reactive metabolites). Recently, a number of articles have been published reporting on the synthesis, biological evaluation, and metabolic profiling of natural/synthetic potential of drug molecules [4,5,6,7,8,9,10,11,12,13,14]. Galeon (Figure 1), a natural cyclic-diarylheptanoid (CDH), was first isolated from *Myrica gale* L. in 1976 by Malterud et al. [13], and many other research groups have reported it from different plant species [11,12,15,16,17,18,19,20,21,22], showing it to have potent biological activities; cytotoxicity against human cancer cell lines A549 (IC_50_ = 2.2 µg/mL) [11], HL-60 (60.33 µmol/L), HeLa (58.21 µmol/L), Hepg2 (63.34 µmol/L) [21], T47D, MCF7, HCT15, and HeLa; anti-tubercular activity against *Mycobacterium tuberculosis* H37Rv in vitro (MIC = 15.0 µg/mL) [18]; chemo-preventive potential [23]; moderate topoisomerase inhibitory activity [4]. Its intriguing molecular structure and various biological properties have led to the establishment of several synthetic methods [4,24,25,26,27,28], which made it possible to pursue its metabolic as well as other additional studies during the development process of the new drug. Recently, we synthesized a series of linear/cyclic-diarylheptanoids (CDHs) and confirmed their cytotoxicity and topoisomerase inhibitory activity [4,6]. As a continuing interest on cyclic diarylheptanoids [4,6,28], we herein report, firstly, in silico prediction of the metabolites of galeon, a natural cyclic-diarylheptanoid (CDH), using the CYP450 module of the StarDrop software [29,30,31], where it used its WhichP450 model to inform us which CYP450 isoforms were the most likely to be responsible for the Phase I metabolism of a compound, and combined with the models of regioselectivities of metabolism by each isoform, the WhichP450 model aided in the prediction of the metabolites most likely to be formed by P450-mediated metabolism. Secondly, we report the metabolite alert sites and toxicity prediction using the Deductive Estimation of Risk from Existing Knowledge (DEREK) software [32,33,34,35], which uses a knowledge-based expert system to predict the toxicity and metabolism of a chemical, facilitating the possibilities of faster scientific progress and reductions in the use of animals in testing [36]. Thirdly, we report on galeon’s metabolic profile through in vitro metabolic experiments using rat liver microsomes (RLMs) in which identification of possible metabolites and confirmation of their structures were carried out using Ion-trap tandem mass spectrometry (LC-MS/MS). Finally, we conducted molecular docking analysis [37] in order to identify the binding mechanism of galeon with all CYP450 isoforms.

## 2. Results and Discussion

### 2.1. In Silico Prediction of Galeon Metabolites Using the CYP450 Metabolism Module of the StarDrop^®^ Software 

**Figure 2:**
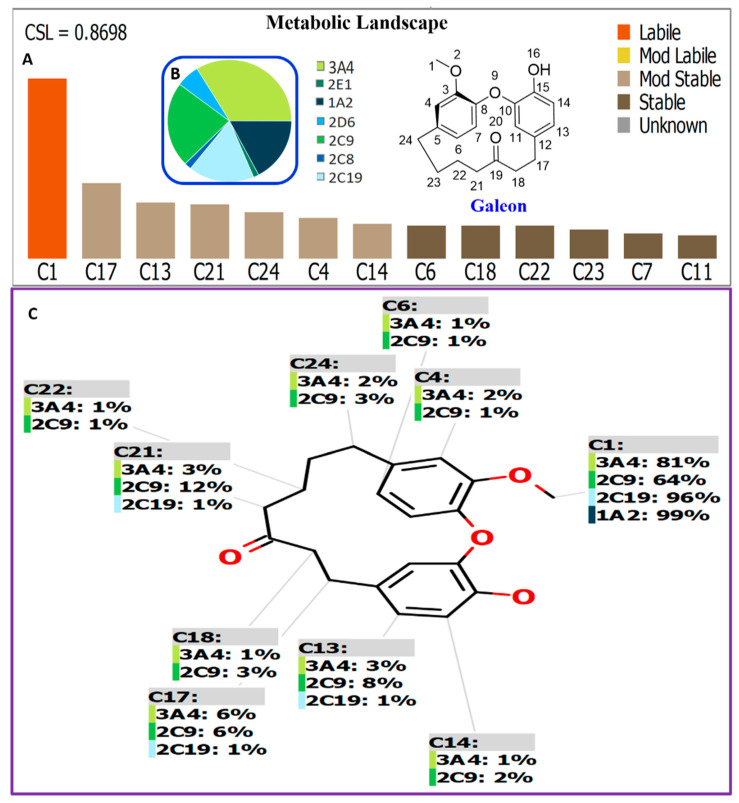
Proposed metabolic sites of galeon using the StarDrop WhichP450™ model: (**A**) Graph showing labile metabolic position; (**B**) pie chart showing enzyme effect in %; (**C**) summary for the major metabolic site (in %) showing the enzymes’ effect. (The pictured was inserted directly from the StarDrop WhichP450™ software).

The vulnerability of key metabolic sites of galeon, indicated by composite site lability (CSL) in a descending manner, is summarized in Figure 2A. The “metabolic landscape” suggested that C1 is the most susceptible site of metabolism, whereas C17, C13, C21, C24, C4, and C14 were predicted to be the next most vulnerable sites of metabolism. The effect of major drug metabolizing CYP450 isoforms (3A4, 2E1, 1A2, 2D6, 2C9, 2C8, and 2C19) on galeon metabolism is summarized in % in a pie chart (Figure 2B), giving information on which isoforms are the most likely to be responsible for Phase I metabolism of galeon. A regio-selectivity map in Figure 2C indicates the possible CYP450 isoforms involved in each site of galeon metabolism. 

**Figure 3:**
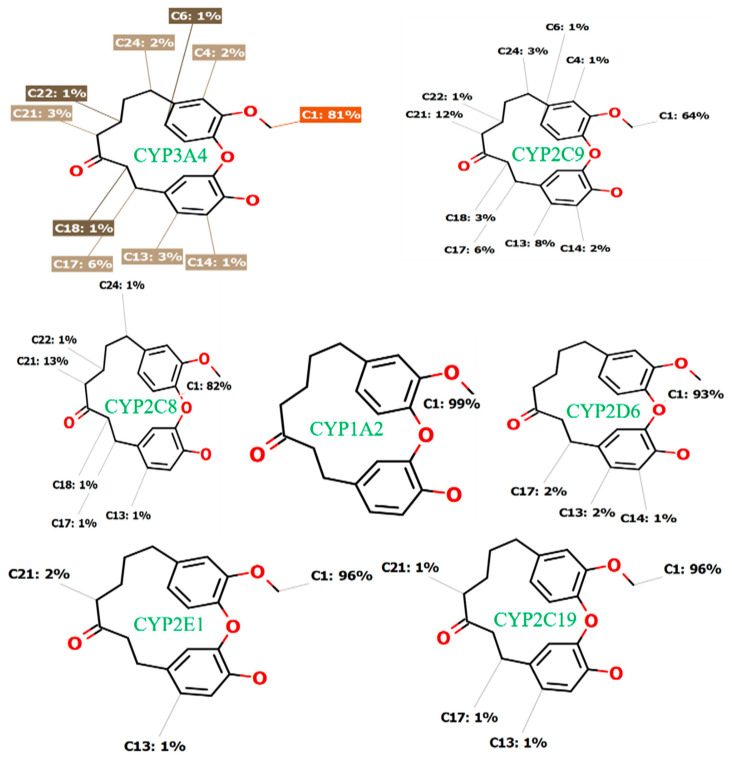
In silico results for CYP450 subtypes affecting galeon. (The picture was inserted directly from the StarDrop WhichP450™ software).

The detailed in silico results for the CYP450 isoforms affecting the metabolism of galeon are illustrated in Figure 3. As displayed, 1A2, 2E1, 2C19, and 2D6 played the major roles in galeon metabolism at the carbon 1 (C1) position, which gave the predicted de-methylated metabolite of galeon, while CYP3A4 was involved in the metabolism on most carbon sites. Being able to generate all the necessary information above, the StarDrop WhichP450™ model aided in the prediction of the metabolites most likely to be formed by CYP450-mediated metabolism so as to guide us to optimize the chemical structure for improving metabolic stability without compromising the efficacy of the drug and with minimal side effects.

### 2.2. In Silico Galeon Structural Alert Sites and Toxicity Prediction

Screening for the predicted toxicity of galeon metabolites was performed using the DEREK software. Galeon and its metabolites’ structural alerts are listed in Table 1. Galeon did not show any structural alerts while the other six predicted mono-hydroxy metabolites of galeon showed skin sensitization, thyroid toxicity, chromosome damage, and carcinogenicity due to the two hydroxyl groups in the same benzene ring forming 1,2-dihydroxybenzene derivatives (catechol) and 1,3-dihydroxybenzene (resorcinol). Table 1 displays a complete list of in vitro galeon metabolites with the DEREK results for proposed toxicity profile.

### 2.3. In Vitro Metabolic Profiling of Galeon 

#### 2.3.1. Mass Spectrometry of Galeon 

**Figure 4:**
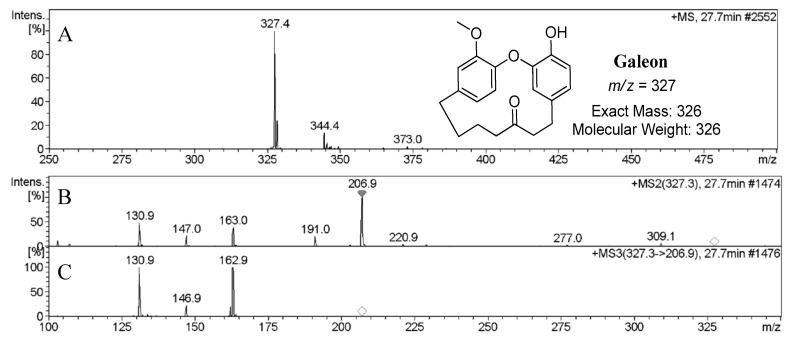
Mass spectra of galeon: (**A**) MS spectrum; (**B**) MS^2^ spectrum; (**C**) MS^3^ spectrum.

A preliminary fragmentation study of galeon was performed in an ion-trap instrument prior to incubating with RLMs to identify fragmentation patterns of the parent compound. Figure 4A shows a mass spectrum of galeon *m/z* 327 at retention time 27.7 min.

**Scheme 1:**
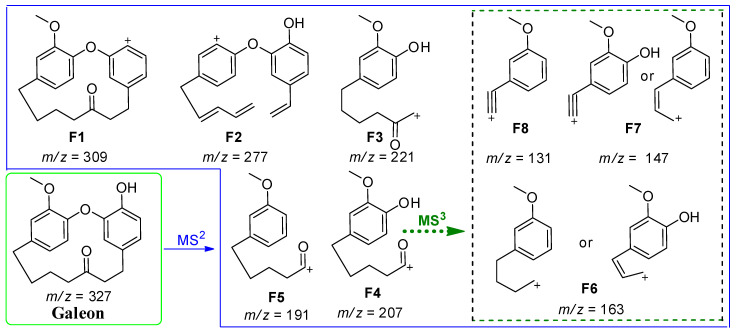
MS/MS^n^ of galeon in ion-trap MS.

The MS^2^ fragmentation of galeon (*m/z* 327) gave major fragments at *m/z* 309 (F1), 277 (F2), 221 (F3), 207 (F4), 191 (F5), 163 (F6), 147 (F7), and 131 (F8), and the MS^3^ fragmentation of the major fragment F4 (*m/z*, 207) gave F6, F7, and F8 (Figure 4B,C). The MS^2^ and MS^3^ fragments and their possible structures are depicted in Scheme 1.

#### 2.3.2. Identification of Phase-I Metabolites

Galeon was incubated with RLMs to attempt to detect Phase-I metabolites in a single run. To investigate reactive metabolite formation, galeon was incubated with RLMs followed by the addition of chemical trapping agents, *N*,*O*-dimethylhydroxylamine (MeNHOCH_3_) and glutathione (GSH), to form stable adducts that can be detected by MS. Three different types of metabolic reactions (i.e., demethylation, mono/di-hydroxylation, and reduction) occurred while incubating galeon, yielding eighteen metabolites in the presence of NADPH-generating system. No metabolites were generated in the absence of either NADPH or microsomes, indicating the likely involvement of CYP450 enzymes and the cofactor NADPH in the metabolisms. The putative metabolites are listed in Table 2 with their *m/z* values, retention times, fragments, and the name of the proposed metabolic reactions. The MS/MS spectra of those metabolites are given in Supplementary Materials Figures S1–S7.

**Scheme 2:**
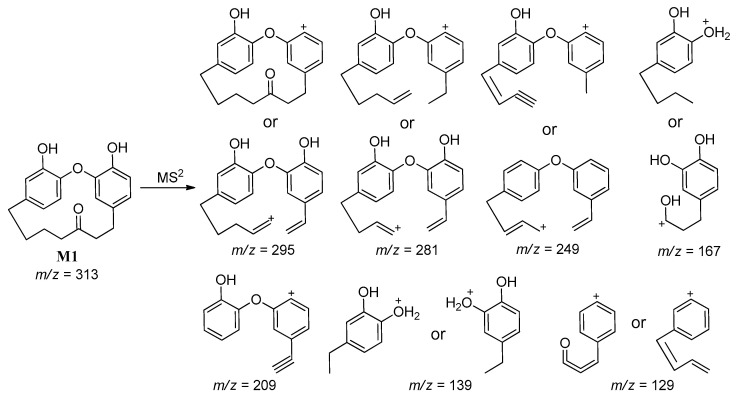
MS^2^ fragmentation pattern of metabolite **M1** (*m/z* 313).

The metabolite **M1** was tentatively identified as a demethylated metabolite of galeon at *m/z* 313. As shown in Table 2, a protonated molecular ion peak at the retention time 25 min gave *m/z* 313 [M + H]^+^ (Figure S1A) with a mass shift of 14Da compared to the parent galeon (*m/z* 327). The MS^2^ experiment of **M1** gave several fragments at *m/z* 295, 281, 249, 209, 167, 139, and 129. The MS/MS^2^ spectra are shown in Figure S1, and the fragmentation pattern with the possible structure of the metabolite are proposed in Scheme 2. Among the fragments, *m/z* 295, 281, and 209 matched with the fragmentation pattern of galeon, and it is only possible if the proposed metabolite structure has a free –OH group instead of –OCH_3_ group. So, the fragmentation pattern supported **M1** as a demethylated metabolite of galeon. 

**Scheme 3:**
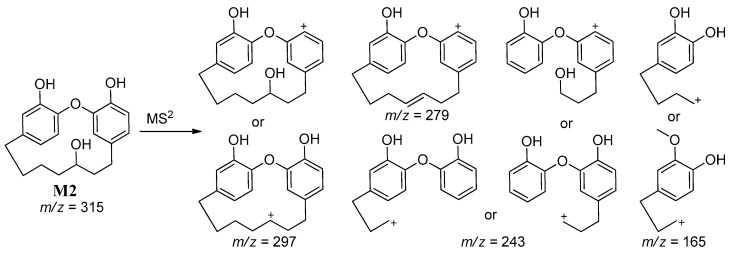
MS^2^ fragmentation pattern of metabolite **M2** (*m/z* 315).

The metabolite **M2** was tentatively identified as demethylated, reduced metabolite of galeon at *m/z* 315. A protonated molecular ion peak at the retention time 28.9 min gave *m/z* 315 [M + H]^+^ (Figure S2A) with a mass shift of 12 Da compared to the parent, where demethylation and reduction occurred. The MS^2^ experiment of **M2** gave fragments at *m/z* 297, 279, 243, and 165. The MS/MS^2^ spectra are shown in Figure S2, and the fragmentation pattern with the possible structure of the metabolite is proposed in Scheme 3. Among the fragments, *m/z* 297 and 165 matched with the fragmentation pattern of galeon. A dehydrated (–H_2_O, 18 Da) ion from 297 at *m/z* 279 is highly possible if the structure of the compound is **M2**.

**Scheme 4:**
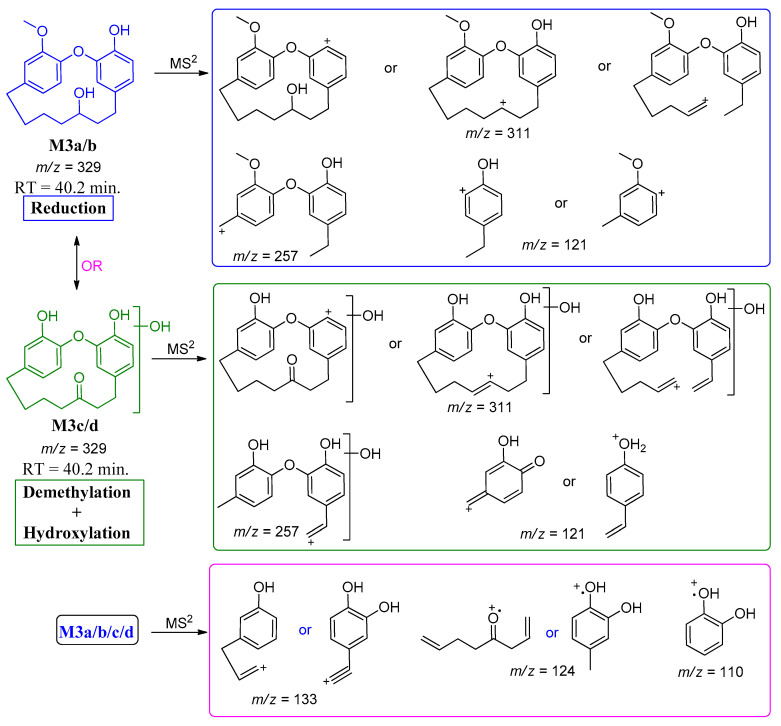
MS^2^ fragmentation pattern of metabolite **M3** (*m/z* 329).

The metabolites **M3a/b/c/d** are tentatively identified as reduced and/or demethylated with hydroxylated metabolites of galeon at *m/z* 329. Protonated molecular ion peaks at retention times 40.0 and 40.6 min gave *m/z* 329 [M + H]^+^ (Figure S3A–C) with a mass shift of 2 Da compared to the parent, where reduction or hydroxylation with demethylation happened in galeon. The MS^2^ experiment of **M3a/b/c/d** at a retention time of 40.0 min gave fragments at *m/z* 311, 257, and 121 (Figure S3B,D). The MS/MS^2^ spectra are shown in Figure S3, and corresponding fragments with possible metabolites structures are depicted in Scheme 4. Among the fragments, *m/z* 311 and 121 matched with the fragmentation pattern of galeon. Thus, the fragmentation pattern supported that the metabolites **M3a/b/c/d** structures’ were the same as depicted in Scheme 4. It should be noted that MS^2^ of *m/z* 329 at the retention time 40.6 min gave fragments at *m/z* 133, 124, and 110, which indicated that metabolites **M3a/b/c/d** can be generated either through reduction or demethylation with hydroxylation.

**Scheme 5:**
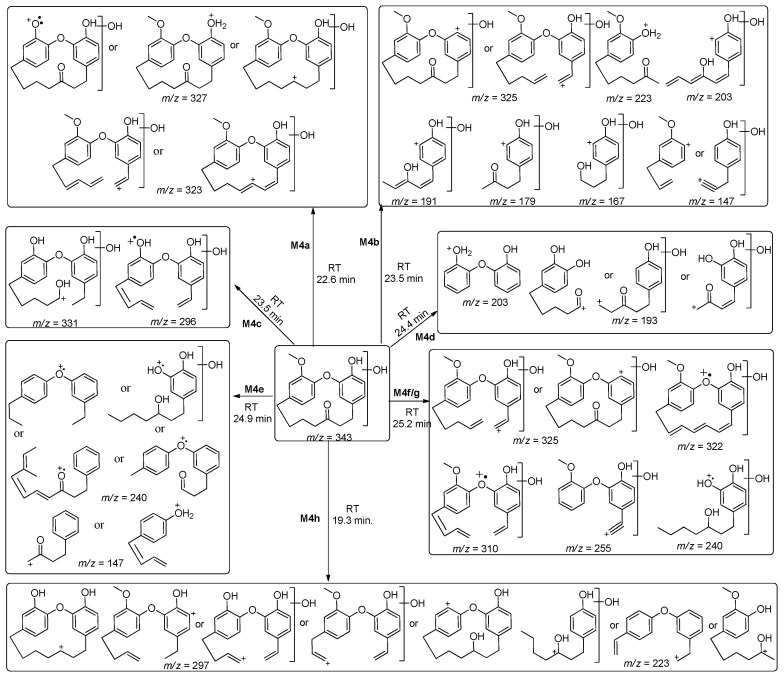
MS^2^ fragmentation pattern of metabolite **M4** (*m/z* 343).

The metabolites **M4a**–**h** were tentatively identified as mono-hydroxylated metabolites of galeon at *m/z* 343. Eight peaks were detected which showed protonated molecular masses at *m/z* 343 [M + H]^+^ with a mass shift of 16 Da from the parent and at the retention times 22.6 min (**M4a**, Figure S4(aA)), 23.5 min (**M4b**, Figure S4(bA)), 24.0 min (**M4c**, Figure S4(cA)), 24.4 min (**M4d**, Figure S4(dA)), 24.9 min (**M4e**, Figure S4(eA)), 25.2 min (**M4f/g**, Figure S4(f/gA)), and 19.3 min (**M4h**, Figure S4(hA)), respectively. The MS^2^ experiments of *m/z* 343 [M + H]^+^, at each corresponding retention time, were performed and different fragments were obtained at *m/z* 327 and 323 (**M4a**, Figure S4(aB)), *m/z* 325, 223, 203, 191, 179, 167, and 147 (**M4b**, Figure S4(bB)), *m/z* 331 and 296 (**M4c**, Figure S4(cB)), *m/z* 203 and 193 (**M4d**, Figure S4(dB)), *m/z* 240 and 147 (**M4e**, Figure S4(eB)), *m/z* 322, 310, 255, and 240 (**M4f/g**, Figure S4(f/gB)); one isomer at 25.2 min), and *m/z* 325, 322, 310, and 240 (**M4g/f**, Figure S4(f/gC)); another isomer at the same retention time), *m/z* 297 and 223 (**M4h**, Figure S4(hB)), respectively. Based on the MS spectra and MS^2^ fragmentation pattern, it was observed that those fragments were only possible if the corresponding compounds were the same as the expected mono-hydroxy metabolites (**M4a**–**h**) of the galeon with their possible structures depicted in Scheme 5.

**Scheme 6:**
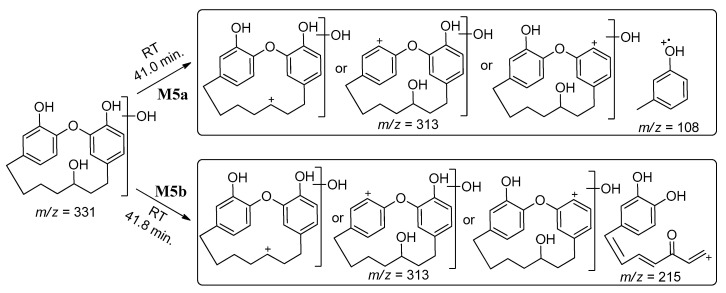
MS^2^ fragmentation pattern of metabolite **M5** (*m/z* 331).

The metabolites **M5a/b** were tentatively identified as demethylated, reduced with hydroxylated metabolite of galeon at *m/z* 331. Protonated molecular ion peaks at the retention times 41.0 min and 41.8 min gave an *m/z* 331 [M + H]^+^ (Figure S5(aA), Figure S5(bA)) with a mass shift of 4 Da compared to the parent. The MS^2^ experiments of *m/z* 331 at 41.0 min and 41.8 min gave fragments at *m/z* 313 and 108 for **M5a** (Figure S5(aB)) and *m/z* 313 and 215 for **M5b** (Figure S5(bB)), respectively. The MS/MS^2^ spectra are shown in Supplementary Materials Figure S5a,b, and the fragmentation patterns with possible structures of the metabolites are proposed in Scheme 6. Those fragments for **M5a** and **M5b** were only possible if the corresponding compounds were produced by the proposed metabolic reactions. 

**Scheme 7:**
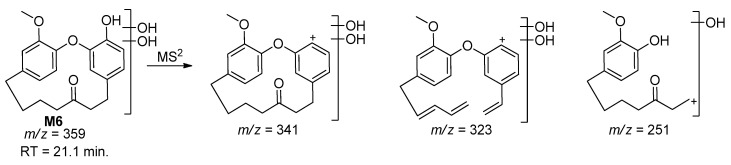
MS^2^ fragmentation pattern of metabolite **M6** (*m/z* 359).

The metabolite **M6** was tentatively identified as a dihydroxylated metabolite of galeon at *m/z* 359. A protonated molecular ion peak at the retention time 21.1 min gave *m/z* 359 [M + H]^+^ (Figure S6A) with mass shift of 32 Da compared to the parent. The MS^2^ experiment of **M6** at *m/z* 359 gave fragments at *m/z* 341, 323, and 251 (Figure S6B). The MS/MS^2^ spectra are shown in Figure S6 and the fragmentation pattern with a possible structure of the metabolite are proposed in Scheme 7. These fragments are only possible if the structure is the same as depicted for **M6**. 

**Scheme 8:**
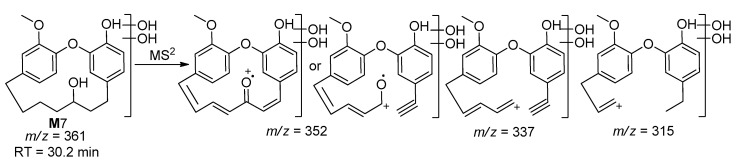
MS^2^ fragmentation pattern of metabolite **M7** (*m/z* 361).

The metabolite **M7** was tentatively identified as reduced, dihydroxylated metabolite of galeon at *m/z* 361. A protonated molecular ion peak at the retention time 30.2 min gave *m/z* 361 [M + H]^+^ (Figure S7A) with a mass shift of 34 Da compared to the parent. The MS^2^ experiment of **M7** at *m/z* 361 gave fragments at *m/z* 352, 337, and 315 (Figure S7B). The MS/MS^2^ spectra are shown in Figure S7, and fragmentation patterns with possible structures of the metabolite are proposed in Scheme 8, and these fragments are only possible if the structure is same as depicted for **M7**. 

**Scheme 9:**
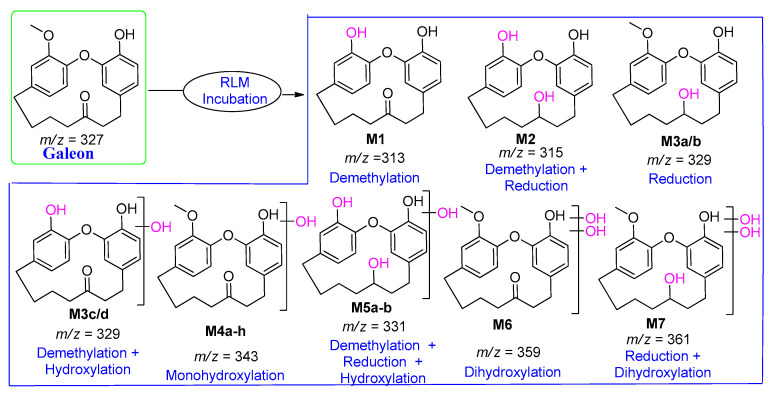
Possible detected metabolic reactions and their proposed structures are depicted. Rat liver microsome, (RLM).

Based on the mass spectra and fragmentation pattern of the identified metabolites, possible metabolic reactions of galeon and a tentative structure of identified metabolites are depicted in Scheme 9. 

As shown in the schemes, there were three types of possible metabolic reactions (i.e., demethylation, reduction, and mono/di-hydroxylation) that occurred in galeon incubation with a total of eighteen metabolites: demethylated (**M1**, *m/z* 313); demethylated with carbonyl reduced (**M2**, *m/z* 315); carbonyl reduced only (**M3a/b**, *m/z* 329) and/or demethylated with hydroxylated (**M3c/d**, *m/z* 329); mono-hydroxylated (**M4a**–**h**, *m/z* 343); demethylated with reduced and mono-hydroxylated (**M5a**–**b**, *m/z* 331); di-hydroxylated (**M6**, *m/z* 359), and di-hydroxylated with reduced (**M7**, *m/z* 361).

#### 2.3.3. Identification of Reactive Metabolites

In the second part of the metabolic study, we tried to identify the possible reactive metabolites by incubating galeon with RLMs in the presence of 2.5 mM *N,O*-dimethylhydroxylamine (MeNHOCH_3_) and 1.0 mM GSH, respectively. Following the extraction of the incubation mixtures, 10 µL of the samples were injected into an LC-MS/MS instrument for analysis. Based on the peak area of the formed metabolites, the hydroxylation pathway was proposed to be the major metabolic pathway for galeon after demethylation or with demethylation. A total of nine conjugates with four types of methoxylamine conjugates and three GSH conjugates were detected (Table 3).

**Scheme 10:**
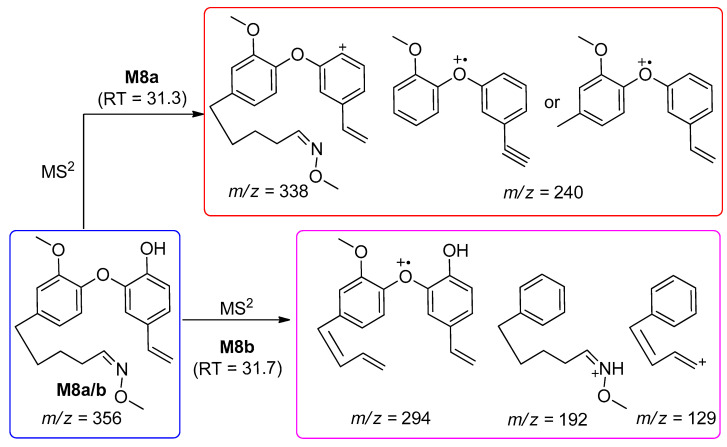
MS^2^ fragmentation pattern of metabolite **M8** (*m/z* 356).

The metabolites **M8a/b** were tentatively identified as oxidative alkylation followed by oxime formation metabolite of galeon at *m/z* 356. Protonated molecular ion peaks at the retention times 31.3 min and 31.7 min gave *m/z* 356 [M + H]^+^ (Figure S8A,C), with a mass shift of 29 Da compared to the parent. The MS^2^ experiments of **M8a** and **M8b** at *m/z* 356 gave fragments at *m/z* 338 and 240 for **M8a** (Figure S8B) and *m/z* 294, 192, and 129 for M8b (Figure S8D), respectively. Based on the fragmentation pattern, a possible structure of the metabolites is proposed in Scheme 10, and these fragments are only possible if the structures are the same as depicted for **M8a/b**. 

**Scheme 11:**
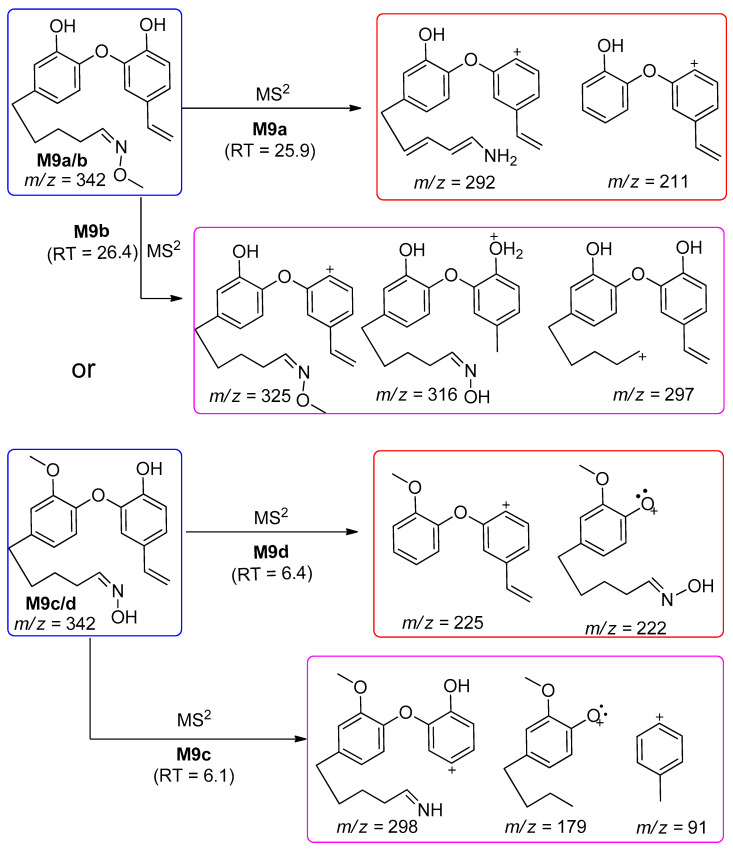
MS^2^ fragmentation pattern of the metabolite **M9** (*m/z* 342).

The metabolites **M9a/b** or metabolites **M9c/d** were tentatively identified as oxidative alkylation, demethylation followed by oxime formation or oxidative alkylation followed by oxime formation, demethylation in oxime metabolite of galeon at *m/z* 342. Protonated molecular ion peaks at the retention times 25.9 min and 26.4 min gave *m/z* 342 [M + H]^+^ (Figure S9(a/bA,a/bC)) with a mass shift of 15 Da compared to the parent. The MS^2^ experiments of **M9a/b** at 25.9 min and 26.4 min at *m/z* 342 gave fragments at *m/z* 292 and 211 (Figure S9(a/bB)) and *m/z* 325, 316, and 297 (Figure S9(a/bD)), respectively. Another set of peaks with the same molecular mass (*m/z* 342 [M + H]^+^) was observed at 6.1 and 6.4 min, which possibly were isomers of **9a/b** and **9c/d** (Figure S9(c/dA)) having a free oxime group. Fragmentation of each peak gave fragments at *m/z* 298, 179, and 91 (Figure S9(c/dB)) and *m/z* 225 and 222 (Figure S9(c/dC)), respectively. Based on the fragmentation pattern, the possible structures of the metabolites are proposed in Scheme 11, and these fragments are only possible if the structures are the same as depicted for **M9a**–**d**. 

**Scheme 12:**
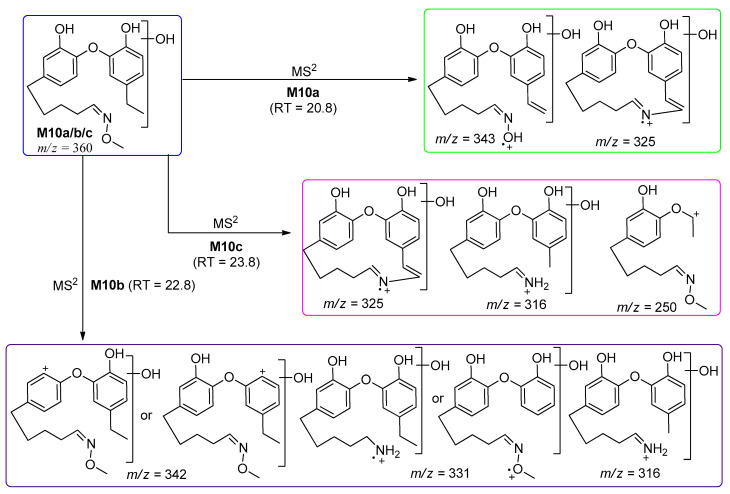
MS^2^ fragmentation pattern of metabolite **M10** (*m/z* 360).

The metabolites **M10a/b/c** were tentatively identified as oxidative alkylation followed by oxime formation, demethylation, and hydroxylation in galeon at *m/z* 360. Protonated molecular ion peaks at the retention times 20.8, 22.8, and 23.8 min give *m/z* 360 [M + H]^+^ (Figure S10A,C,E) with a mass shift of 33 Da compared to the parent. The MS^2^ experiments of *m/z* 360 at 20.8, 22.8, and 23.8 min gave fragments at *m/z* 343 and 325 (Figure S10B), *m/z* 342, 331, and 316 (Figure S10D), and *m/z* 325, 316, and 250 (Figure S10F), accordingly. Based on the fragmentation pattern, the only possible structures of the metabolites are proposed in Scheme 12. 

**Scheme 13:**
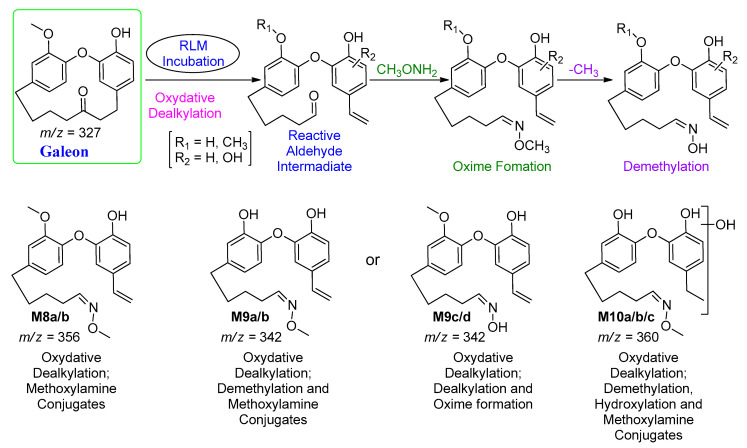
Possible methoxylamine conjugates.

Based on the mass spectra and fragmentation patterns of identified metabolites **M8** to **M10**, possible metabolic reactions of galeon and tentative structures of the identified metabolites are depicted in Scheme 13.

**Scheme 14:**
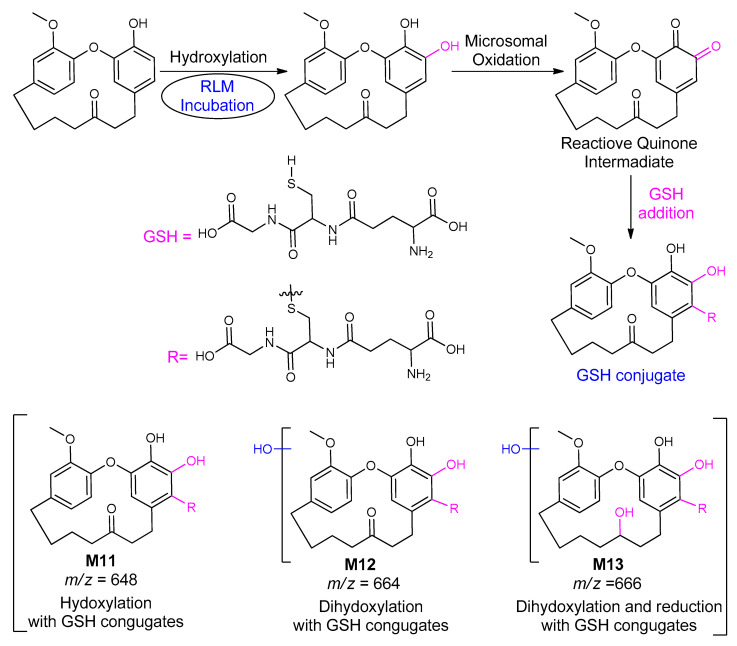
Mechanism of GSH conjugation of galeon.

Screening for GSH adducts in the control galeon incubation without RLMs revealed the absence of GSH peaks in the Total Ion Chromatogram (TIC). Bio-activation of galeon requires an enzyme for the hydroxylation and subsequent oxidation to form the quinone reactive intermediates, which only occurs in the presence of RLMs (Scheme 14).

**Scheme 15:**
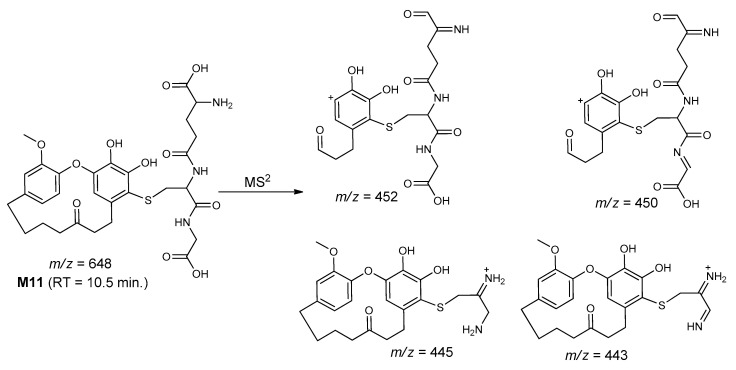
MS^2^ fragmentation pattern of metabolite **M11** (*m/z* 648).

The metabolite **M11** was tentatively identified as hydroxylation followed by GSH conjugation of galeon at *m/z* 648. A protonated molecular ion peak at the retention time 10.5 min gave *m/z* 648 [M + H]^+^ (Figure S11A) with a mass shift of 323 Da compared to the parent, where hydroxylation followed by GSH conjugation took place. The MS^2^ experiment of **M11** at *m/z* 648 gave fragments at *m/z* 452, 450, 445, and 443 (Figure S11C). Based on the fragmentation pattern, these fragments were only possible if the structure of the metabolite was the same as proposed in Scheme 15, indicating the confirmation of the GSH conjugate of galeon (**M11**). 

**Scheme 16:**
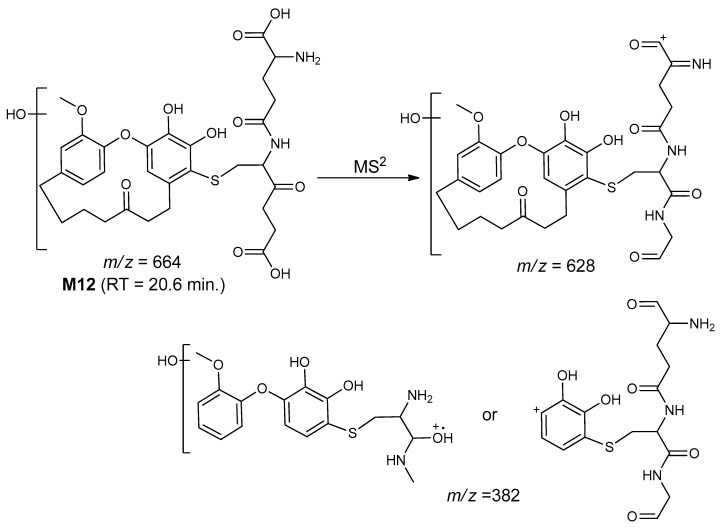
MS^2^ fragmentation pattern of metabolite **M12** (*m/z* 664).

The metabolites **M12** was tentatively identified as dihydroxylation followed by GSH conjugation of galeon at *m/z* 664. A protonated molecular ion peak at the retention time 20.6 min gave *m/z* 664 [M + H]^+^ (Figure S12A) with mass shift of 337 Da compared to the parent, where dihydroxylation followed by GSH conjugation occurred. The MS^2^ experiment of **M12** at *m/z* 664 gave fragments at *m/z* 628 and 382 (Figure S12B). Based on the fragmentation pattern, these fragments were only possible if the structure of the metabolite **M12** was the same as proposed in Scheme 16, indicating the confirmation of the GSH conjugate of galeon (**M12**). 

**Scheme 17:**
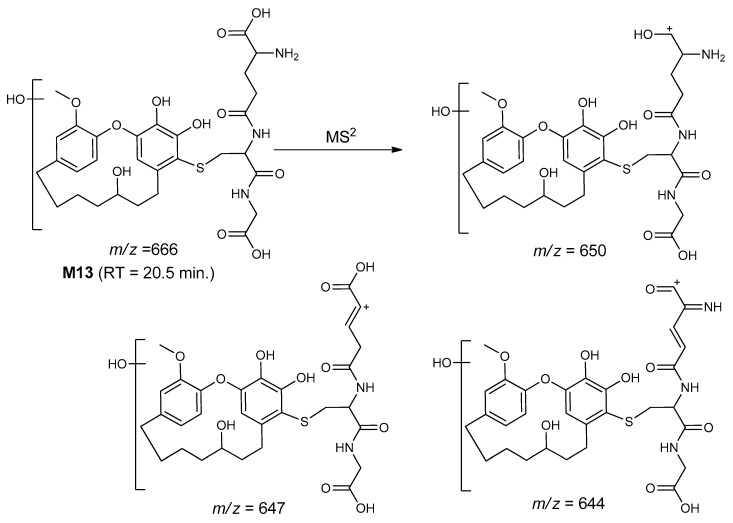
MS^2^ fragmentation pattern of metabolite **M13** (*m/z* 666).

The metabolite **M13** was tentatively identified as dihydroxylation and reduction followed by GSH conjugation of galeon at *m/z* 666. A protonated molecular ion peak at the retention time 20.5 min gave *m/z* 666 [M + H]^+^ (Figure S13A) with a mass shift of 339 Da compared to the parent, where dihydroxylation and reduction followed by GSH conjugation occurred. The MS^2^ experiment of **M13** at *m/z* 666 gave fragments at *m/z* 650, 647, and 644 (Figure S13B). Based on the fragmentation pattern, theses fragments were only possible if the structure of the metabolite **M13** was the same as proposed in Scheme 17, indicating the confirmation of the GSH conjugate of galeon (**M13**).

### 2.4. In Silico Binding Mechanism of Galeon with Cytochrome P450 Isoforms

The in silico metabolism analysis of galeon, as shown in Figure 2 and Figure 3, indicate that the C1 position was the most vulnerable metabolic site of galeon (Figure 2A), and CYP450 isoforms led to a variable range of metabolisms starting from 64% to 99% (Figure 3), where the CYP2C9 isoform gave the least (64%) and CYP1A2 gave the highest (99%) metabolism. In order to understand how galeon interacts with the CYP450 isoforms to lead to such a different range of metabolism, in silico docking analysis for the interaction of galeon with CYP450 isoforms was performed. Therefore, only the isoforms with more than 90% metabolism profiling were selected, excluding those below 90%, except for CYP3A4, as the software predicted its involvement in various metabolic reactions at various target sites in galeon.

**Figure 5:**
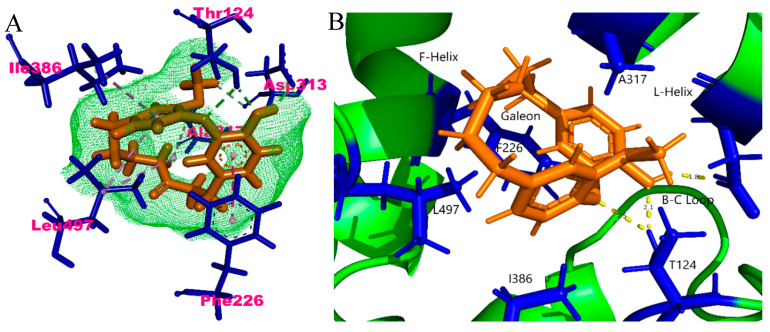
(**A**) 2D interaction mechanism of CYP1A2–galeon; (**B**) 3D interaction mechanism of CYP1A2–galeon.

Firstly, in silico docking analysis was carried out for the CYP1A2 isoform which showed the maximum metabolic profiling (99%) prediction. We found that galeon interacts with the active site of CYP1A2 via the Thr124, Phe226, Asp313, Ala317, Ile386, and Leu497 residues (Figure 5A). Among these residues, Thr124 and Asp313 formed conventional hydrogen bonds with the –OH group at the number 16 position of galeon. Additionally, Phe226 formed π–π stacked bond, while Ala317, Ile386, and Leu497 formed π–alkyl interactions. Analyzing the crystal structure of CYP1A2 isoforms, it was evident that Phe226 from Helix-F produced a substrate binding surface as well as a Ala317 side chain from Helix-L and other residues forming the binding cavity [38]. Further hydrogen bonding interactions with the side chain of Thr223 on Helix-F with the side chain of Asp320 on Helix-L, connecting both helices at the roof of the cavity. Both Thr223 and Asp320 provide an extensive network of interactions supporting the active site. Although galeon missed some important residue interactions of the CYP1A2 active site, the docking study revealed clear interactions of galeon with Phe226 of Helix-F and Ala317 of Helix-L (Figure 5A,B), which is enough to support the in silico metabolic profiling with only one metabolic target site in galeon by the CYP1A2 isoform. 

**Figure 6:**
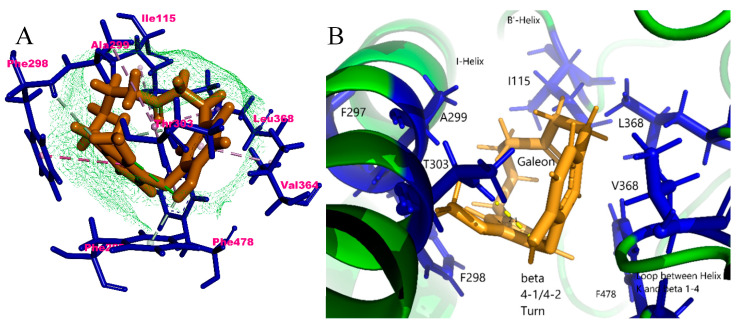
(**A**) 2D interaction mechanism of CYP2E1-galeon; (**B**) 3D interaction mechanism of CYP2E1-galeon.

Secondly, galeon was docked with the CYP2E1 isoform which showed a 96% metabolic profiling, and the results are shown in Figure 6. Galeon and CYP2E1’s interaction involved Ile115, Phe207, Phe298, Ala299, Thr303, Val364, Leu368, and Phe478 residues (Figure 6A). The amino acid residues Phe207 and Thr303 were involved to form π–donor hydrogen bonds interactions with H of the –OH group; Phe298 and Phe478 by π–π T-shaped interactions; Ile115, Ala299, Val364, and Leu368 by π–alkyl interactions. According to CYP2E1’s crystal structure [39], the main binding site for ligands were Phe106, Phe116, Ile115, and Val364. Galeon would, thus, interact with Ile115 among these residues of the active site. Thr303’s polar side chain lining the active site cavity was highly conserved which interacted with galeon via hydrogen bonding. This residue may play a similar role in the positioning of smaller substrates. Evidence suggests that this conserved Thr303 plays an important role in proton delivery to the active site in other P-450 enzymes [40,41]. In addition, CYP2E1 [42], Val364, Leu368, and Phe478 would form a constriction between the active site cavity and access channel thereby helping the translocation of the substrate. The location of the main residues involved is depicted in Figure 3, where Ile115 belongs to the B’-Helix, Val364 and Leu368 belong to the loop between Helix-K and beta 1-4, Phe478 belongs to the beta 4-1/4-2 turn, and Thr303 belongs to the Helix-I (Figure 6B).

**Figure 7:**
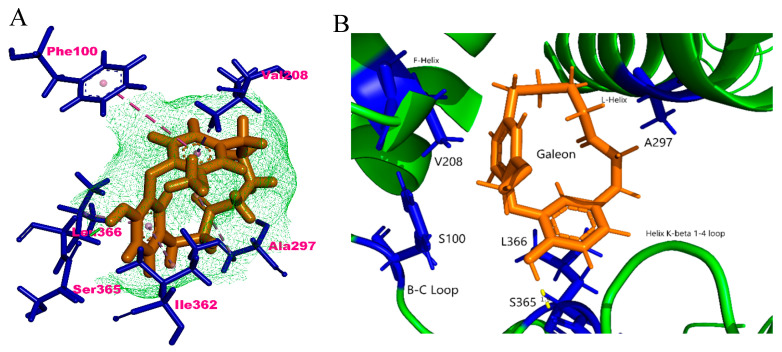
(**A**) 2D interaction mechanism of CYP2C19-galeon; (**B**) 3D interaction mechanism of CYP2C19-galeon.

Next, a docking analysis for galeon with the CYP2C19 isoform with a 96% metabolic profiling is shown in Figure 7. It shows one conventional hydrogen bond interaction of the –OH group with Ser365 residue; three π–alkyl interactions with Ala297, Ile362, and Leu366; a π–π stacked bonding with Phe10; a single van der Waals interaction with Val208 (Figure 7A). Structural analysis from the previously published crystal structure of CYP2C19 revealed important residues responsible for substrate binding and metabolism [39]. The location of the main residues involved is depicted in Figure 6, where Ser365, Ile362, and Leu36 belong to Helix-K/Beta 1-4 and Ala297 belongs to the Helix-L loop of the active site (Figure 7B). Arg108 mainly functions for higher substrate binding affinity, but galeon did not interact with it. Phe100 resides in the B–C loop and Val208 in the Helix-F (Figure 7B). Phe100 performs the open–close mechanism of CYP2C19’s active site by changing the conformation of the B–C loop. The Helix-K/Beta 1-4 loop’s residues help surround the active site’s cavities formed by two separate cavities. However, galeon lacks interactions with Arg108, an important active site residue of CYP2C19, thus showing the lower affinity and fewer metabolic sites to support in silico metabolic profile analyses (Figure 7). 

**Figure 8:**
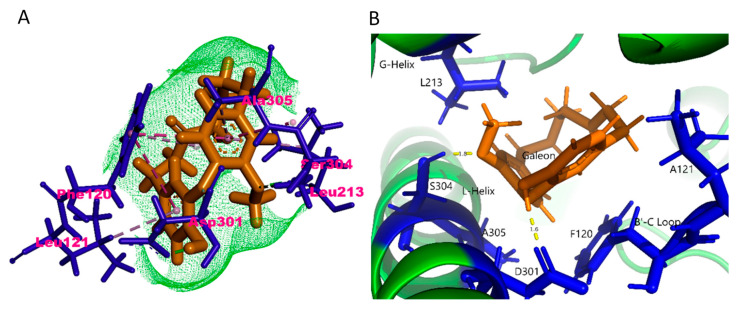
(**A**) 2D interaction mechanism of CYP2D6-galeon; (**B**) 3D interaction mechanism of CYP2D6-galeon.

Docking analysis for galeon with the CYP2D6 isoform with a 93% metabolic profiling is shown in Figure 8. One of the important interaction sites involved between CYP2D6 and galeon is Phe120 in the B’–C loop, which plays key roles for two π–π stacked bonding between the benzene rings, Leu121 in the B’–C loop for π–alkyl interaction, and Leu213 in the G-helix region for one T-shaped π–π bonding. In addition, Asp301 in the L-Helix region was involved in the interaction with the H of –OH group via conventional hydrogen bonding, Ser304 and Ala305 in the L-helix via hydrogen bonding as well as π–π stacked T-shaped π–π interaction (Figure 8A,B). The docking analysis data clearly showed that galeon interacted with the residues mostly present in the L-helix, G-helix, and B’–C loop region. The crystal structure of CYP2D6 suggests that an active site cavity would be formed by residues from the B-helix, the B′–C loop (side chains of Leu-110, Phe-112, Phe-120, and Leu-121 and the main chain atoms of Gln-117, Gly-118, Val-119, and Ala-122), the F-helix (side chains of Leu-213, Glu-216, Ser-217, and Leu-220), the G-helix, the L-helix (side chains of Ile-297, Ala-300, Asp-301, Ser-304, Ala-305, Val-308, and Thr-309), the loop between K-helix and β sheet 1 strand 4, and residues from the loop between the strands of the β sheet 4 [43].

**Figure 9:**
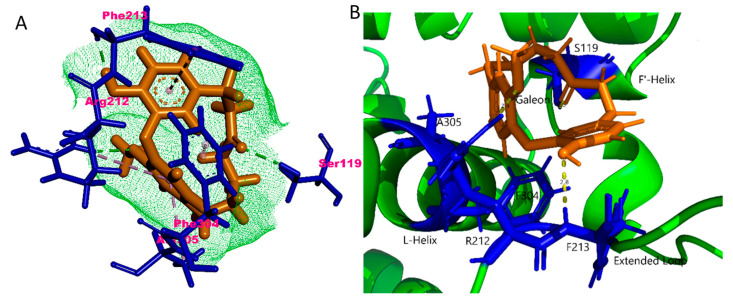
(**A**) 2D interaction mechanism of CYP3A4-galeon; (**B**) 3D interaction mechanism of CYP3A4-galeon.

Finally, a docking analysis was performed for galeon with the CYP3A4 isoform with an 81% metabolic profile (Figure 9). It should be noted that, the CYP3A4 isoforms are not present in the rat liver microsomes (RLMs) but in the human liver microsomes (HLMs). Galeon interacted with Ser119 of CYP3A4 in the F-helix via hydrogen bonding with =O group, Arg212 in the L-helix by H-bonding and pi-alkyl bonding, respectively, with the –OCH3 group and the corresponding benzene ring, Phe213, in the extended loop by H-bonding and T-shaped π–π bonding, respectively, with the –OH group and the corresponding benzene ring, Phe304, in the L-helix via π–alkyl interaction with the center of the compound, and Ala305 in the L-helix by π–alkyl interaction with benzene ring of the –OCH3 group. Structural analysis revealed that [44] Phe213 and Phe215 form the active site and, together with another five phenylalanine residues (Phe108, Phe219, Phe220, Phe241, and Phe304), form a “Phe-cluster”, which have also been shown by mutagenesis studies to be involved in CYP3A4 activity. Ala305 is highly conserved and functionally important for enzymatic activity. Galeon successfully made important interactions with Phe-cluster residues thus showing the highest metabolic sites within galeon and proving to be a superior metabolic substrate (Figure 9A,B).

Overall, galeon showed significant interactions with active site residues of approximately all CYP450 isoforms, although CYP1A2 and CYP2C19 showed to be less active metabolically against galeon due to the lack of important interactions with the active site residues. Interestingly, CYP3A4 and CYP2E1 gave the best metabolic activity against galeon. The CYP2D6 isoform on the other hand showed a measurable amount of enzymatic activity against galeon. 

**Figure 10:**
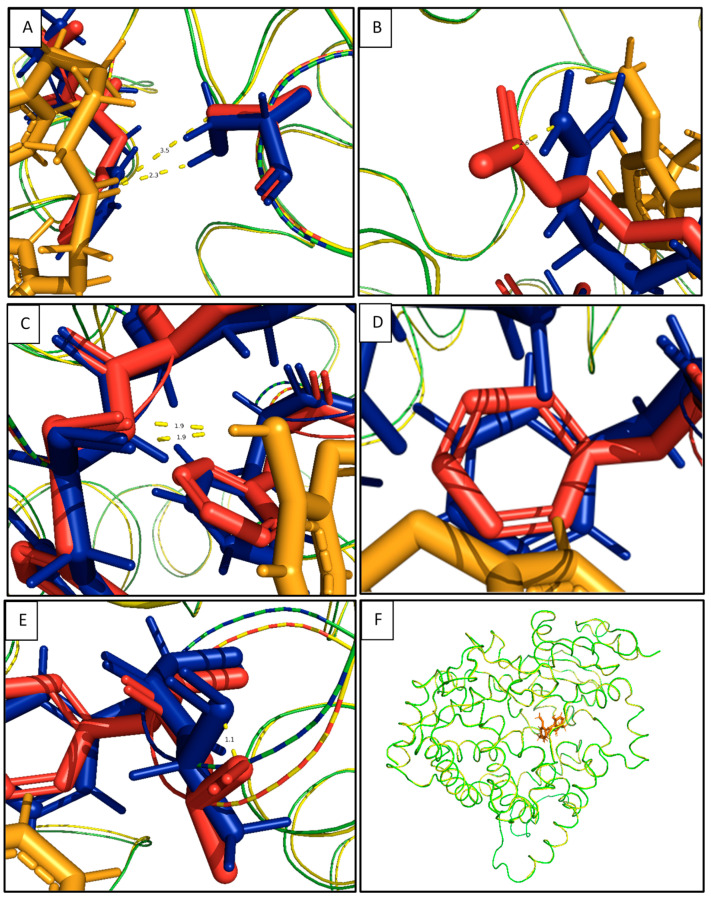
Conformational changes of the interacting residues: (**A**) Ser119, (**B**) Arg212, (**C**) Phe213, (**D**) Phe304, (**E**) Ala305, and (**F**) the overall structural alignment. The Apo state is represented in yellow, while the ligand bound state is shown in green. The Apo state interacting residues are in red, and ligand bound state residues are in blue.

From the docking analysis, we found that the CYP3A4 isoform showed the highest metabolic activity against galeon. We analyzed the conformational difference in the interacting residues of the active site between the apo and ligand bound states. The overall root-mean-square deviation (RMSD) between the apo and galeon bound states was 0.163 Å of 438 C∝ atoms. When analyzing the active site, Ser119 moved inward to the galeon by approximately 1 Å (Figure 10A). In addition, Arg212 gave a clockwise rotation of 2.6 Å towards galeon, making it possible for interaction to occur (Figure 10B). However, Phe213 did not show any significant change in both the apo and ligand bound states (Figure 10C). Similarly, Phe304 lacked inward or outward movement, but showed a slight rotatory movement among themselves (Figure 10D). On the other hand, Ala305 showed approximately a 1Å anti-clockwise rotation (Figure 10E). The overall structural alignment between the apo and galeon bound states can be found in Figure 10F.

## 3. Materials and Methods 

### 3.1. In Silico Study

Structural modification of galeon to reduce their side effects was proposed using the StarDrop software. The result from the StarDrop WhichP450™ model, shown using a pie chart, was used for the indication of the most likely CYP450 isoform that played a major role in galeon metabolism. The DEREK software was utilized to screen for structural alerts to confirm our bio-activation proposal and predicted toxicity of galeon metabolites. In order to identify the binding mechanism of galeon with all CYP450 isoforms, molecular docking was carried out using a previously reported method [37]. The protein structures of the corresponding CYP450 isoforms were downloaded from the Research Collaboratory for Structural Bioinformatics (RCSB) Protein Data Bank (PDB) database (https://www.rcsb.org/) in PDB format. The PDB IDs used for the isoforms were 2HI4, 3E4E, 4GQS, 2F9Q, and 1W0G for CYP1A2, CYP2E1, CYP2C19, CYP2D6, and CYP3A4, respectively. Proteins and ligands were prepared for docking by the established procedure [37]. Structural alignment of apo and galeon bound states of CYP3A4 was performed using Pymol.

### 3.2. Chemicals, Reagents, and Instrumentation 

Synthesized galeon was used for metabolic experiments [4]. Rat liver microsomes (RLMs) were obtained from our colleague, Mohamed W. Attwa, College of Pharmacy, KSU. Mass spectrometric data analysis was performed in a 1200 series LC (Agilent Technologies) coupled with Agilent 6320 ion trap MS fitted with electrospray ionization (ESI) ion source. Column: Eclipse XDB-C18 (150 × 4.6 mm, 5 µm). Gradient solvent system: acetonitrile (ACN) and water (H_2_O) (Table S2). Flow rate: 0.3 mL/min, run time: 60 min (Table S2). Temperature of the capillary 350 °C; flow rate of dry gas was 10.0 L/min; nebulizer pressure: 60 psi; maximum accumulation time was 100,000 μs. The capillary voltage was set at 1.50 V. The ion source was ESI and the capillary voltage was 4000 V. Spectra were taken in the positive/negative modes, and the scan range was 50–700 *m/z*; the injection volume was 1–10 μL.

### 3.3. RLMs Incubations

Metabolism of galeon (1 µL of 1 mM stock solution; 0.326 mg/mL in DMSO) was determined in 40 µL (2 mg/mL) of RLMs in potassium phosphate buffer (0.08 M KH_2_PO_4_/NaH_2_PO_4_, pH 7.4) at 37 °C for 30 min, with freshly prepared ice-cold MgCl_2_ (20.33 mg/mL) solution (final incubation volume was 1 mL). The incubation mixtures were allowed to stand at 37 °C in the shaking water bath for 5 min, before adding NADPH (0.1 mL; 0.8 mM; 8.33 mg/mL) to the incubation mixture to initiate the reaction. For reactive metabolites incubation, galeon with RLMs in the presence of chemical trapping agents 2.5 mM methoxylamine/1.0 mM GSH were added before adding NADPH. Two duplicate tests along with three controls were performed (the values are summarized in Table S1). 

## 4. Conclusions

In silico galeon metabolite analysis revealed demethylated galeon was the most predicted metabolite in addition to mono-hydroxy metabolites. According to the alert site prediction, the isoforms CYP1A2, CYP2E1, CYP2C19, CYP2D6, and CYP3A4 were involved in metabolic profiling. Among the predicted metabolites, mono-hydroxy metabolites were found to have plausible toxicity, due to the formation of 1,2-dihydroxybenzene (catechol) and 1,3-dihydroxybenzene, (resorcinol), in skin sensitization, thyroid toxicity, chromosome damage, and carcinogenicity. From the in vitro metabolic profiling of galeon in RLMs, eighteen Phase-I metabolites, nine methoxylamine, and three GSH conjugates were identified in the presence of NADPH, chemical trapping agents *N,O*-dimethylhydroxylamine, and GSH, respectively. Identification and elucidation of the structures of possible identified metabolites were performed using LC-MS/MS mass and their corresponding fragmentation pattern. In silico docking analysis of galeon showed significant interactions with active site residues of approximately all CYP450 isoforms.

## Figures and Tables

**Table 1:** Qualitative toxicity prediction of galeon and its metabolites by Deductive Estimation of Risk from Existing Knowledge (DEREK) software analysis.

Galeon and Its Metabolites	Skin Sensitization	Thyroid Toxicity	Chromosome Damage	Carcinogenicity	Teratogenicity, Genotoxicity, and Mutagenicity
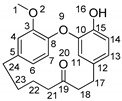 Galeon	NA	NA	NA	NA	NA
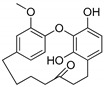 Hydroxy(C11)-galeon	Plausible	NA	NA	NA	NA
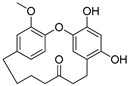 Hydroxy(C13)-galeon	Plausible	Plausible	NA	Plausible	NA
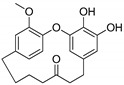 Hydroxy(C14)-galeon	Plausible	NA	Plausible	NA	NA
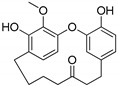 Hydroxy(C4)-galeon	Plausible	NA	NA	NA	NA
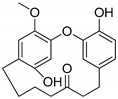 Hydroxy(C6)-galeon	Plausible	NA	NA	Plausible	NA
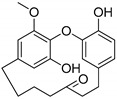 Hydroxy(C7)-galeon	Plausible	NA	NA	NA	NA

NA = Not applicable (i.e., no toxicity).

**Table 2:** Possible Phase-I metabolites of galeon.

Metabolites	Nominal Observed Mass (*m/z*)	Retention Time (min)	Fragments (MS^2^ in *m/z*)	Possible Metabolic Reactions
**M1**	313	25.0	295, 281, 249, 209, 167, 139, 129	Demethylation
**M2**	315	28.9	297, 279, 243, 165	Demethylation with Reduction
**M3a/b or M3c/d**	329	40.0 or 40.6	311, 257, 121 133, 124, 110	Reduction or Demethylation with Hydroxylation
**M4a**	343	22.6	327, 323	Hydroxylation
**M4b**		23.5	325, 223, 203, 191, 179, 167, 147	
**M4c**		24.0	331, 296	
**M4d**		24.4	203, 193	
**M4e**		24.9	240, 147	
**M4f/g**		25.2	322, 310, 255, 240/325, 322, 310, 240	
**M4h**		19.3	297, 223	
**M5a**	331	41.0	313, 108	Demethylation with Hydroxylation and Reduction
**M5b**		41.8	313, 215	
**M6**	359	21.1	341, 323, 251	Dihydroxylation
**M7**	361	30.2	352, 337, 315	Dihydroxylation with Reduction

**Table 3:** Reactive metabolites of galeon.

Metabolites	Nominal Observed Mass (m/z)	Retention Time (min)	Fragments (MS^2^ in m/z)	Metabolic Reactions
Methoxylamine–Galeon Conjugate				
**M8a**	356	31.3	338, 240	Oxidative alkylation followed by oxime formation
**M8b**		31.7	294, 192, 129	
**M9a**	342	25.9	292, 211	Oxidative alkylation and demethylation followed by oxime formation
**M9b**		26.4	324, 316, 297	
**M9c**		6.1	298, 179, 91	
**M9d**		6.4	225, 222	
**M10a**	360	20.8	343, 325	Oxidative alkylation, demethylation and hydroxylation followed by oxime formation
**M10b**		22.8	342, 331, 316	
**M10c**		23.8	325, 316, 250	
Glutathione (GHS)–Galeon Conjugate				
**M11**	648	10.5	452, 450, 445, 443	Mono-hydroxygaleon–GSH conjugate
**M12**	664	20.6	628, 382	Di-hydroxygaleon–GSH conjugate
**M13**	666	20.5	650, 647, 644	Reduction of carbonyl and Di-hydroxygaleon–GSH conjugate

## Supplementary Materials

The following are available online, **Figure S1**: MS/MS^n^ Spectra of metabolite **M1**, Figure S2: MS/MS^n^ Spectra of metabolite **M2**, Figure S3: MS/MS^n^ Spectra of metabolite **M3**, Figure S4a: MS/MS^n^ Spectra of metabolite **M4a**, Figure S4b: MS/MS^n^ Spectra of metabolite **M4b**, Figure S4c: MS/MS^n^ Spectra of metabolite **M4c**, Figure S4d: MS/MS^n^ Spectra of metabolite **M4d**, Figure S4e: MS/MS^n^ Spectra of metabolite **M4e**, Figure S4f/g: MS/MS^n^ Spectra of metabolite **M4f/g**, Figure S4h: MS/MS^n^ Spectra of metabolite **M4h**, Figure S5a: MS/MS^n^ Spectra of metabolite **M5a**, Figure S5b: MS/MS^n^ Spectra of metabolite **M5b**, Figure S6: MS/MS^n^ Spectra of metabolite **M6**, Figure S7: MS/MS^n^ Spectra of metabolite **M7**, Figure S8: MS/MS^n^ Spectra of metabolite **M8**, Figure S9a/b: MS/MS^n^ Spectra of metabolite **M9a/b**, Figure S9c/d: MS/MS^n^ Spectra of metabolite **M9c/d**, Figure S10: MS/MS^n^ Spectra of metabolite **M10**, Figure S11: MS/MS^n^ Spectra of metabolite **M11**, Figure S12: MS/MS^n^ Spectra of metabolite **M12**, Figure S13: MS/MS^n^ Spectra of metabolite **M13**, Table S1: RLMs incubations of Galeon and Table S2: LC Gradient solvent system.
