# Supplementary material for: Galeon: A Biologically Active Molecule with In Silico Metabolite Prediction, In Vitro Metabolic Profiling in Rat Liver Microsomes, and In Silico Binding Mechanisms with CYP450 Isoforms
Source: Molecules. 2020 Dec 13;25(24):5903. doi: 10.3390/molecules25245903 (PMC7763192; doi:10.3390/molecules25245903)
Supplement: Supplementary file 1 [file molecules-25-05903-s001.pdf]

Galeon: A Biologically Active Molecule with In Silico Metabolite Prediction, In Vitro Metabolic Profiling in Rat Liver Microsomes, and In Silico Binding Mechanisms with CYP450 Isoforms

A. F. M. Motiur Rahman,[1,*] Wencui Yin[1], Adnan A. Kadi[1] and Yurngdong Jahng[2]

[1]*Department of Pharmaceutical Chemistry, College of Pharmacy, King Saud University, Riyadh 11451, Saudi Arabia.*

[2]*College of Pharmacy, Yeungnam University, Gyeongsan 38541, Republic of Korea.*

*Corresponding Authors:

Tel: +966114670237, Fax: +966114676220, Email: afmrahman@ksu.edu.sa

Entry	**Content**	Page
Figure-**S1**	MS/MS^n Spectra of metabolite **M1**	3
Figure-**S2**	MS/MS^n Spectra of metabolite **M2**	3
Figure-**S3**	MS/MS^n Spectra of metabolite **M3**	4
Figure-**S4a**	MS/MS^n Spectra of metabolite **M4a**	5
Figure-**S4b**	MS/MS^n Spectra of metabolite **M4b**	5
Figure-**S4c**	MS/MS^n Spectra of metabolite **M4c**	6
Figure-**S4d**	MS/MS^n Spectra of metabolite **M4d**	6
Figure-**S4e**	MS/MS^n Spectra of metabolite **M4e**	7
Figure **S4f/g**	MS/MS^n Spectra of metabolite **M4f/g**	7
Figure-**S4h**	MS/MS^n Spectra of metabolite **M4h**	8
Figure-**S5a**	MS/MS^n Spectra of metabolite **M5a**	8
Figure-**S5b**	MS/MS^n Spectra of metabolite **M5b**	9
Figure-**S6**	MS/MS^n Spectra of metabolite **M6**	9
Figure-**S7**	MS/MS^n Spectra of metabolite **M7**	10
Figure-**S8**	MS/MS^n Spectra of metabolite **M8**	11
Figure-**S9a/b**	MS/MS^n Spectra of metabolite **M9a/b**	12
Figure-**S9c/d**	MS/MS^n Spectra of metabolite **M9c/d**	13
Figure-**S10**	MS/MS^n Spectra of metabolite **M10**	14
Figure-**S11**	MS/MS^n Spectra of metabolite **M11**	15
Figure-**S12**	MS/MS^n Spectra of metabolite **M12**	15
Figure-**S13**	MS/MS^n Spectra of metabolite **M13**	16
Table-**S1**	RLMs incubations of Galeon	17
Table-**S2**	LC Gradient solvent system	18

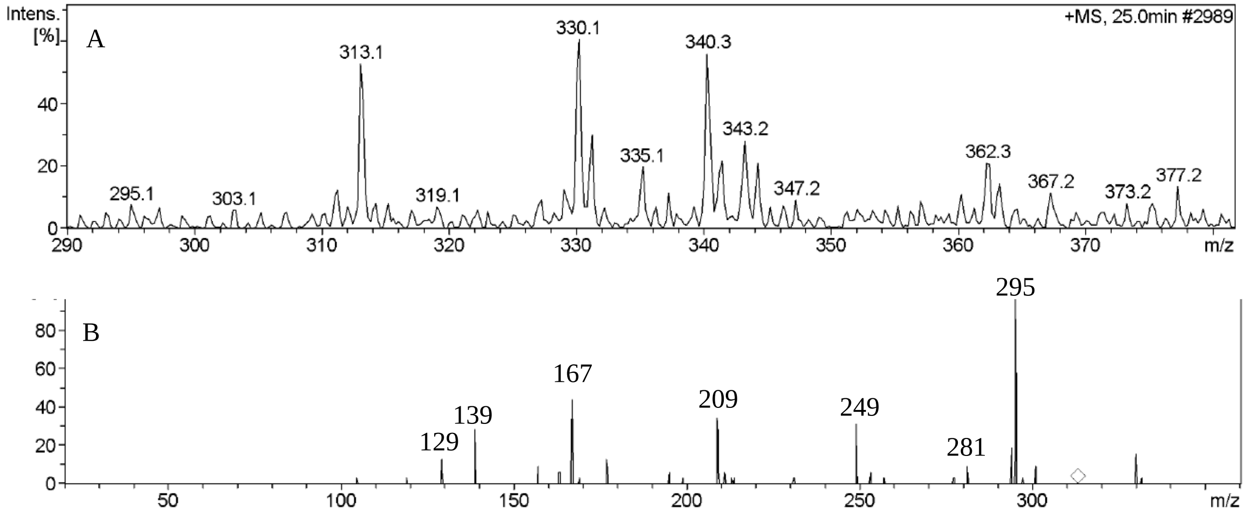

Figure-**S1**: MS/MS^{n} Spectra of **M1**: A) Mass spectrum of **M1** (*m/z* = 313; RT = 25 min.); B) MS^{2} spectrum of **M1** (at *m/z* = 313)

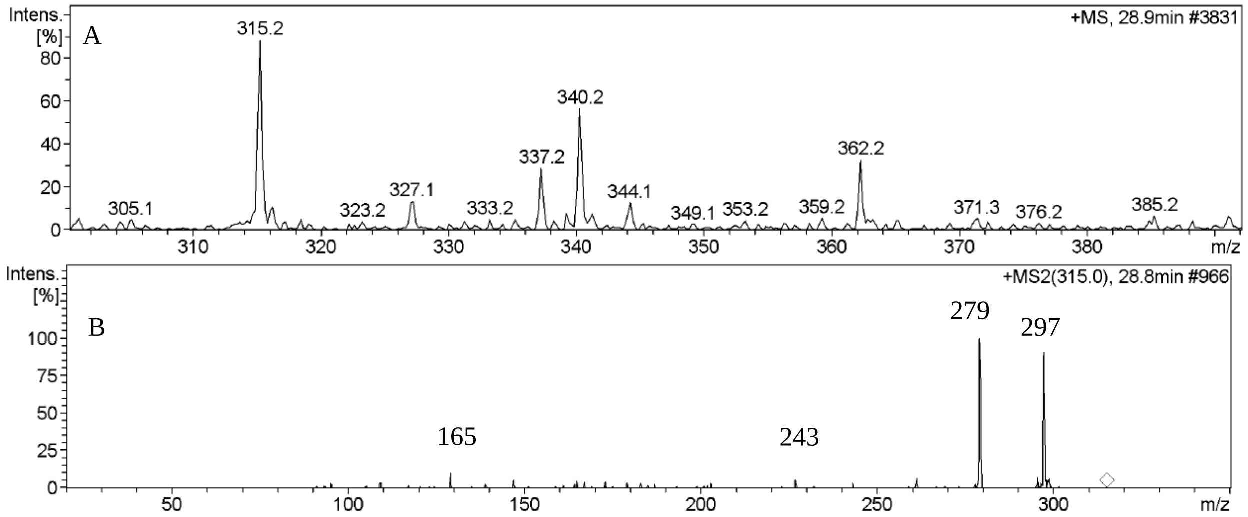

Figure-**S2**: MS/MS^{n} Spectra of **M2**: A) Mass spectrum of **M2** (*m/z* = 315; RT = 28.9 min.); B) MS^{2} spectrum of **M2** (at *m/z* = 315)

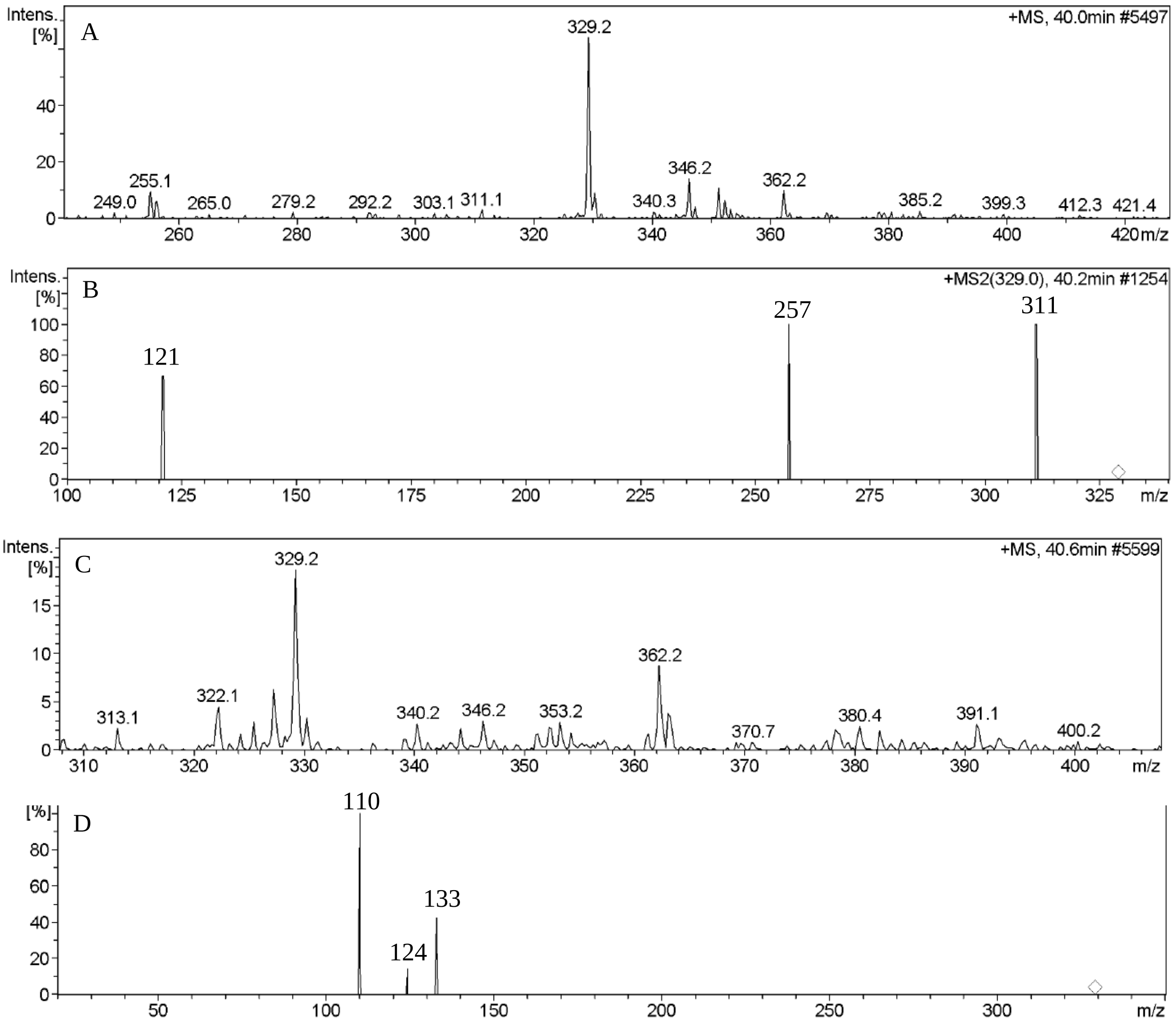

Figure-**S3**. MS/MS^n Spectra of **M3:** A) Mass spectrum of **M3a/b/c/d** (*m/z* = 329; RT = 40.0 min.); B) MS^2 spectrum of **M3a/b/c/d** (at *m/z* = 329); C) Mass spectrum of **M3a/b/c/d** (*m/z* = 329; RT = 40.6 min.); D) MS^2 spectrum of **M3a/b/c/d** (at *m/z* = 329)

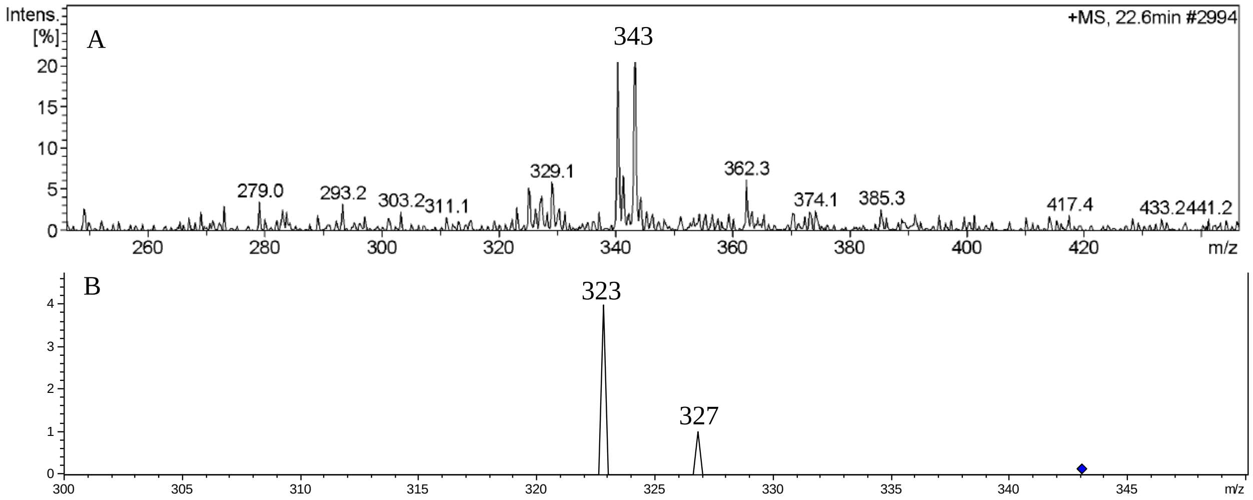

Figure-**S4**. MS/MS^n Spectra of **M4a:** A) Mass spectrum of **M4a** (m/z = 343; RT = 22.6 min.); B) MS^2 spectrum of **M4a** (at m/z = 343)

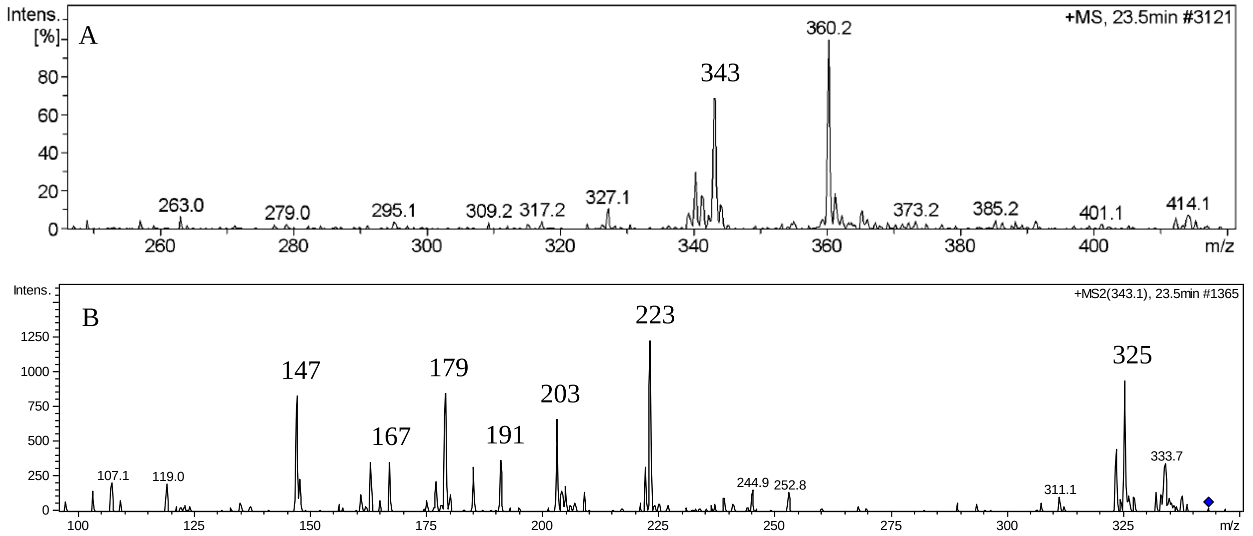

Figure-**S4b**. MS/MS^n Spectra of **M4b:** A) Mass spectrum of **M4b** (m/z = 343; RT = 23.5 min.); B) MS^2 spectrum of **M4b** (at m/z = 343)

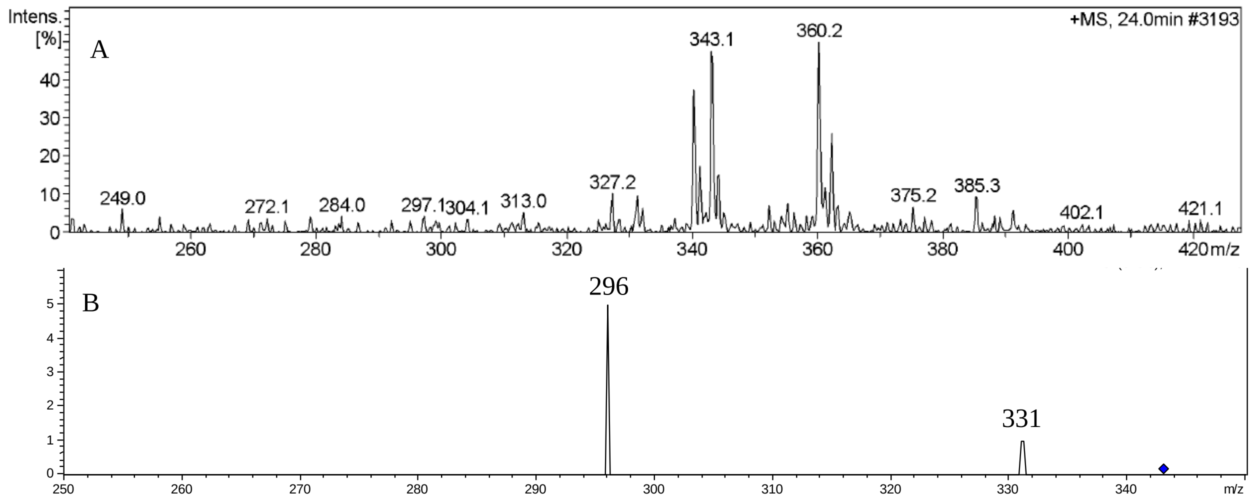

Figure-**S4c**. MS/MS^{n} Spectra of **M4c:** A) Mass spectrum of **M4c** (*m/z* = 343; RT = 24.0 min.); B) MS^{2} spectrum of **M4c** (at *m/z* = 343)

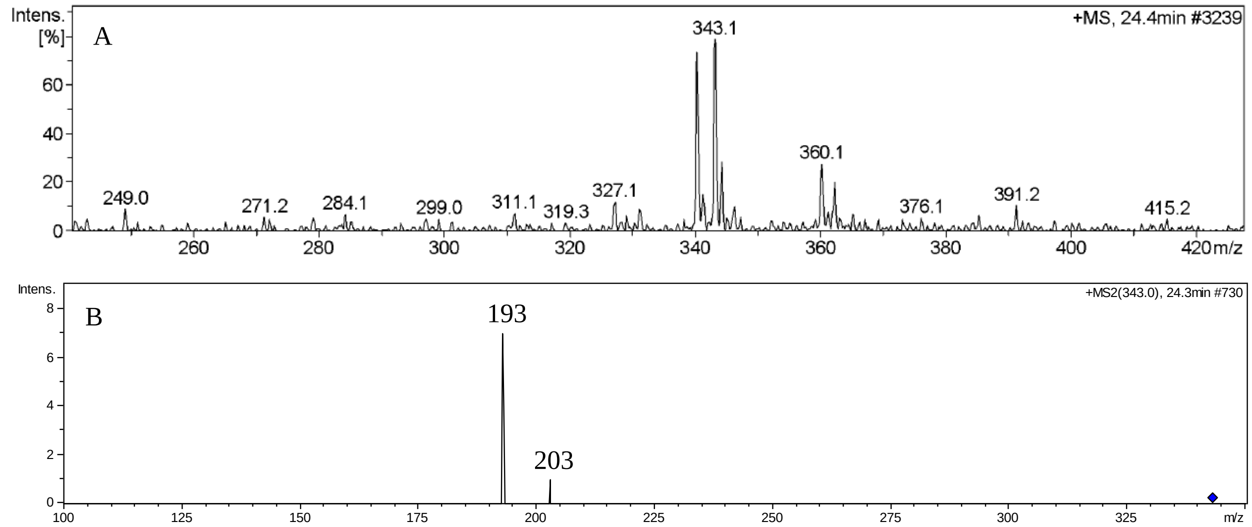

Figure-**S4d**. MS/MS^{n} Spectra of **M4d:** A) Mass spectrum of **M4d** (*m/z* = 343; RT = 24.4 min.); B) MS^{2} spectrum of **M4d** (at *m/z* = 343)

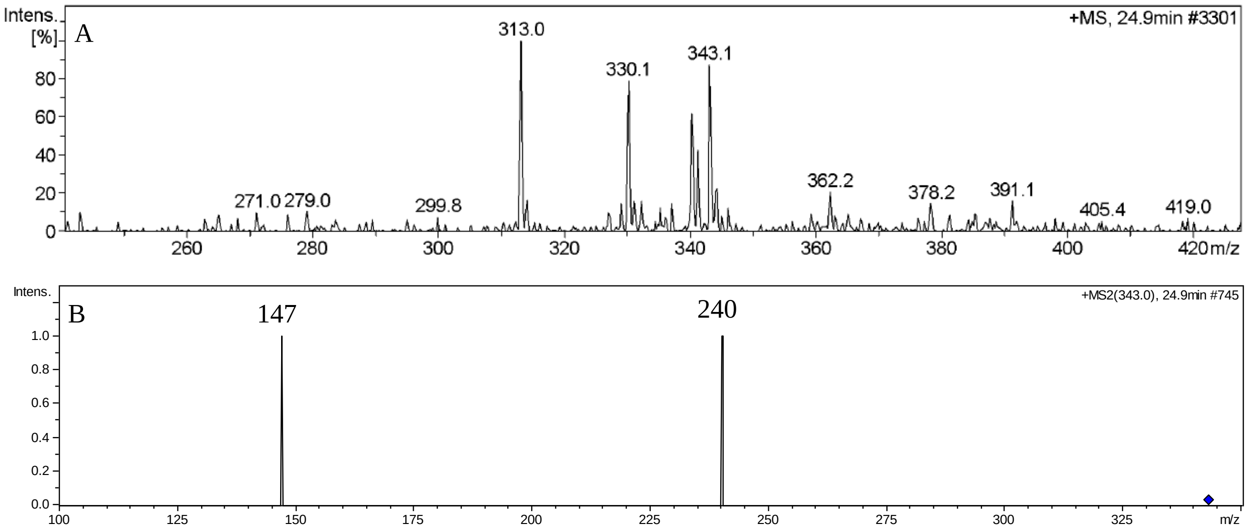

Figure-**S4e**. MS/MS^n Spectra of **M4e:** A) Mass spectrum of **M4e** (*m/z* = 343; RT = 24.9 min.); B) MS^2 spectrum of **M4e** (at *m/z* = 343)

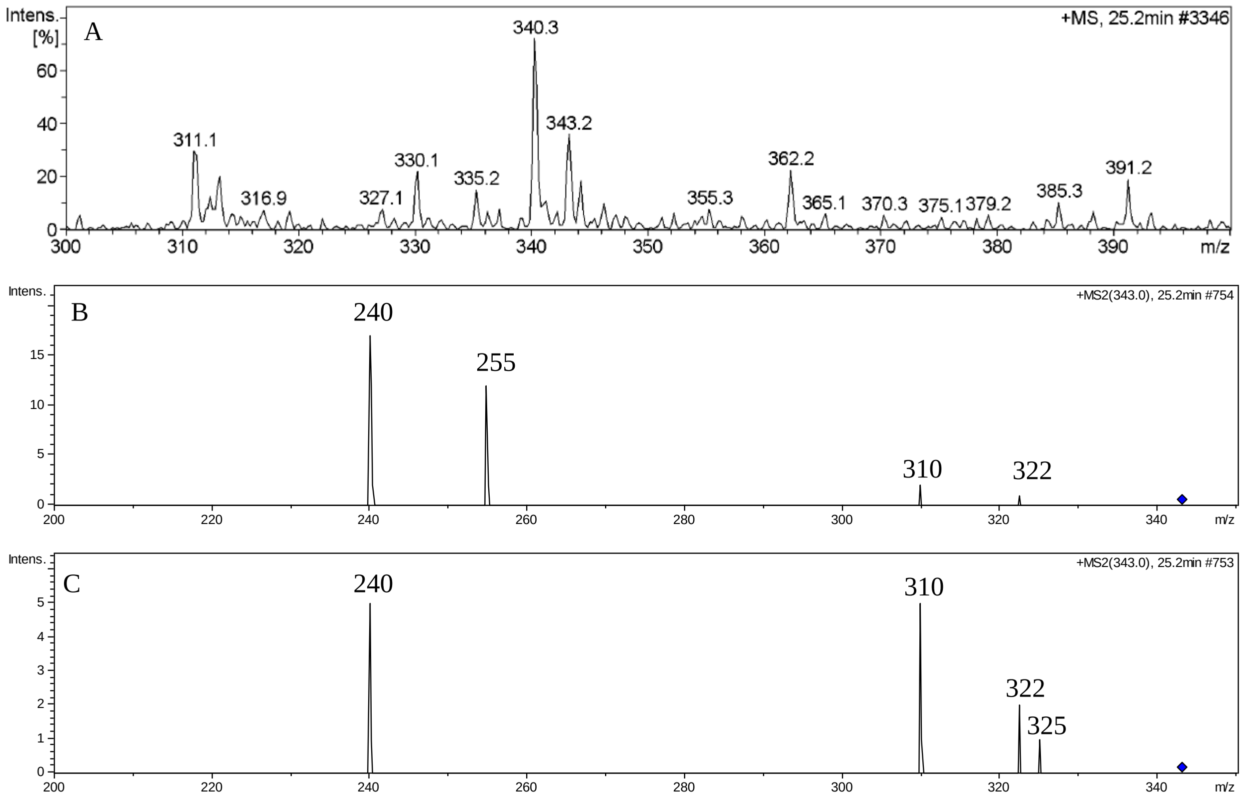

Figure-**S4f/g**. MS/MS^n Spectra of **M4f/g:** A) Mass spectrum of **M4f/g** (*m/z* = 343; RT = 25.2 min.); B&C) MS^2 spectrum of **M4f/g** (at *m/z* = 343)

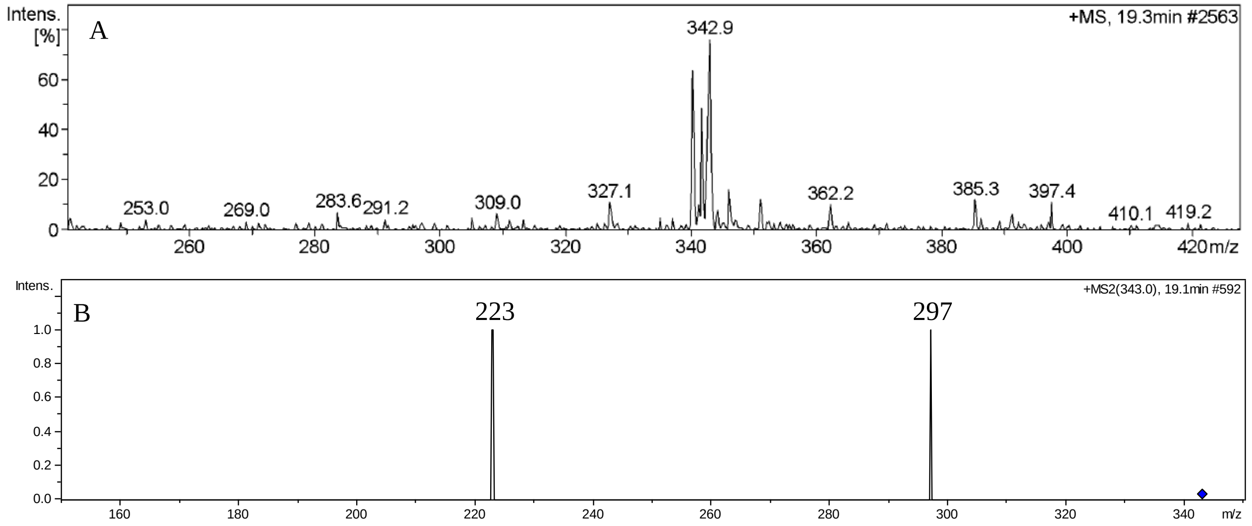

Figure-**S4h**. MS/MS^n Spectra of **M4h:** A) Mass spectrum of **M4h** (*m/z* = 343; RT = 19.3 min.); B) MS^2 spectrum of **M4h** (at *m/z* = 343)

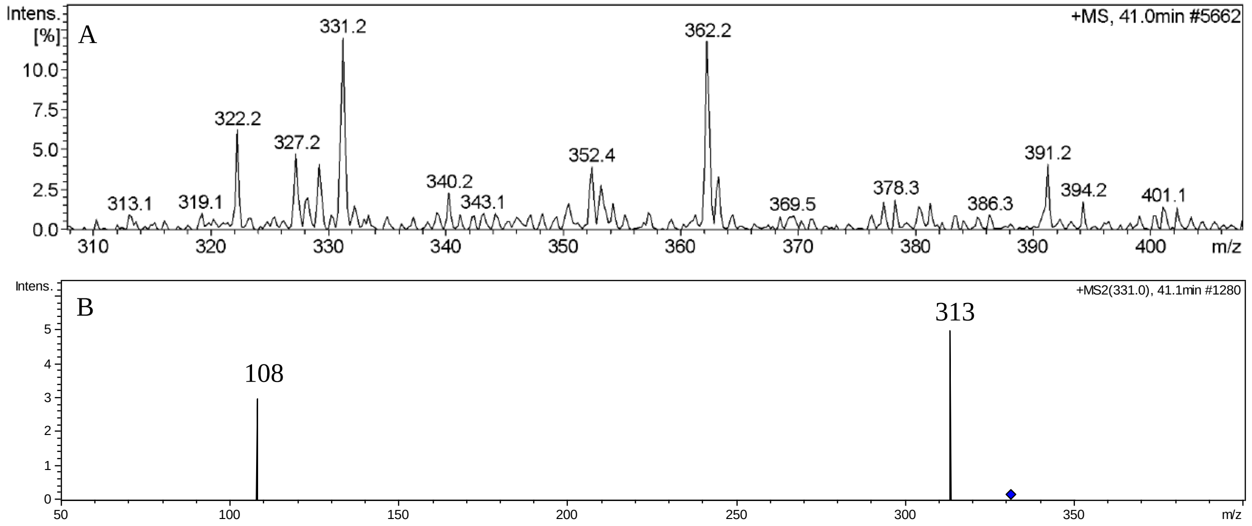

Figure-**S5a**. MS/MS^n Spectra of **M5a:** A) Mass spectrum of **M5a** (*m/z* = 331; RT = 41.0 min.); B) MS^2 spectrum of **M5a** (at *m/z* = 331)

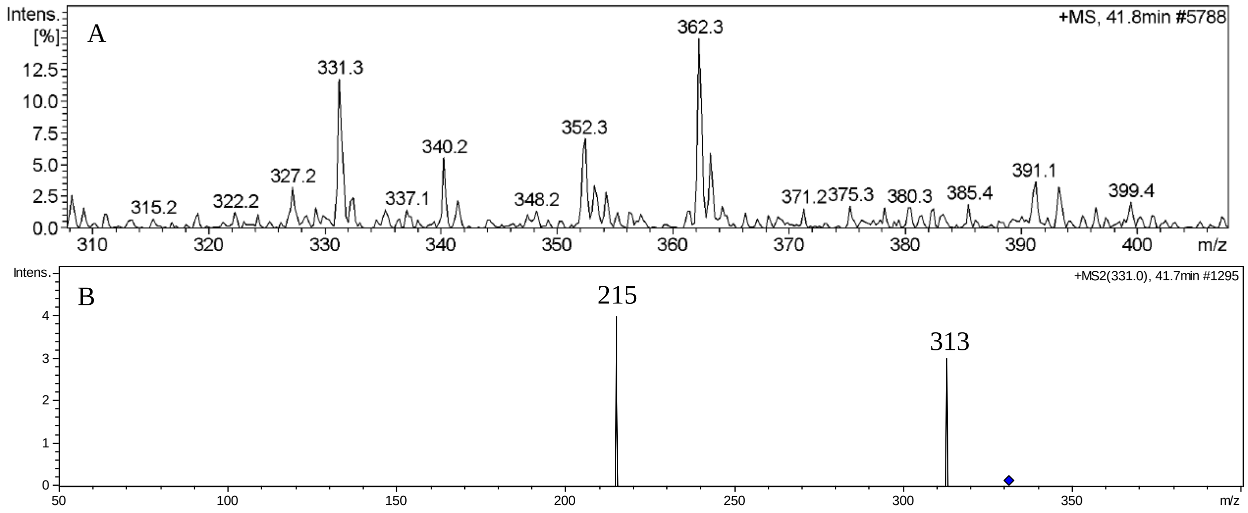

Figure-**S5b**. MS/MS^n Spectra of **M5b:** A) Mass spectrum of **M5b** (*m/z* = 331; RT = 41.0 min.); B) MS^2 spectrum of **M5b** (at *m/z* = 331)

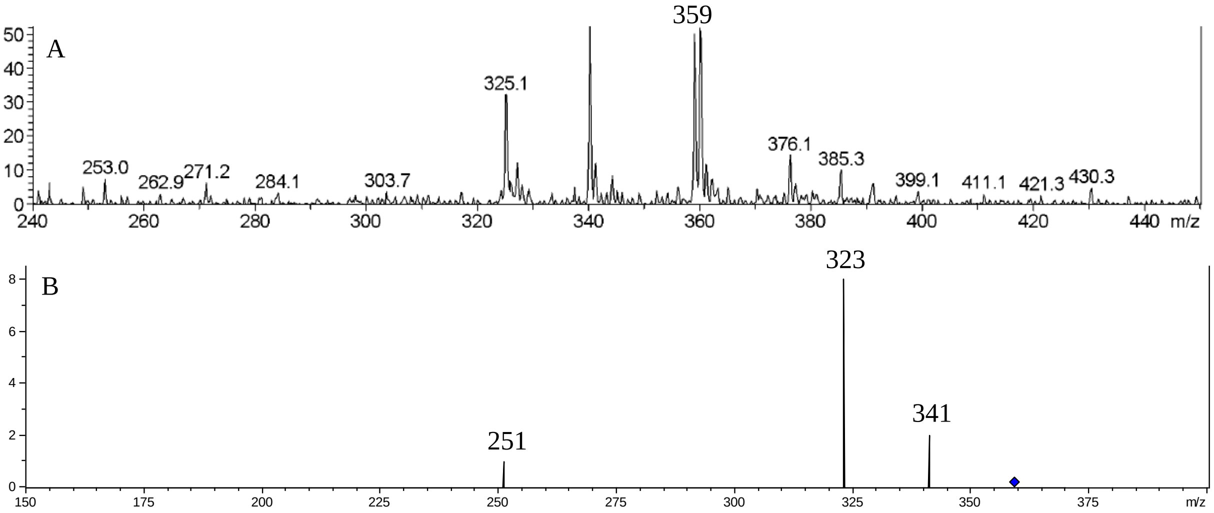

Figure-**S6**: MS/MS^n Spectra of **M6**: A) Mass spectrum of **M6** (*m/z* = 359; RT = 21.1 min.); B) MS^2 spectrum of **M6** (at *m/z* = 359)

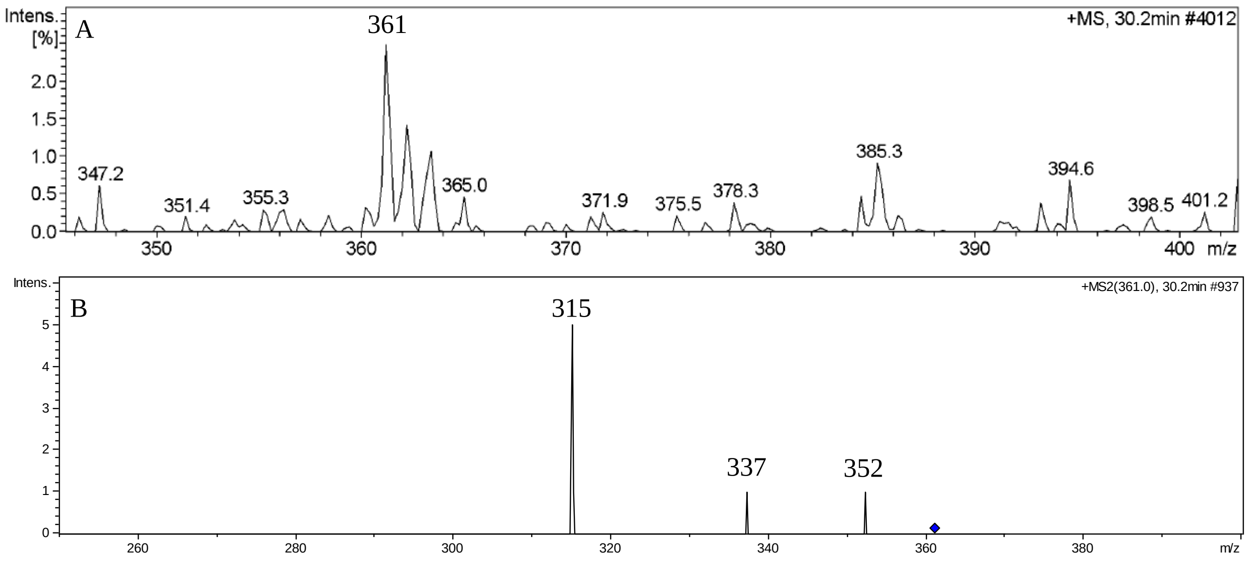

Figure-**S7**: MS/MS^n Spectra of **M7**: A) Mass spectrum of **M7** (m/z = 361; RT = 30.2 min.); B) MS^2 spectrum of **M7** (at m/z = 361)

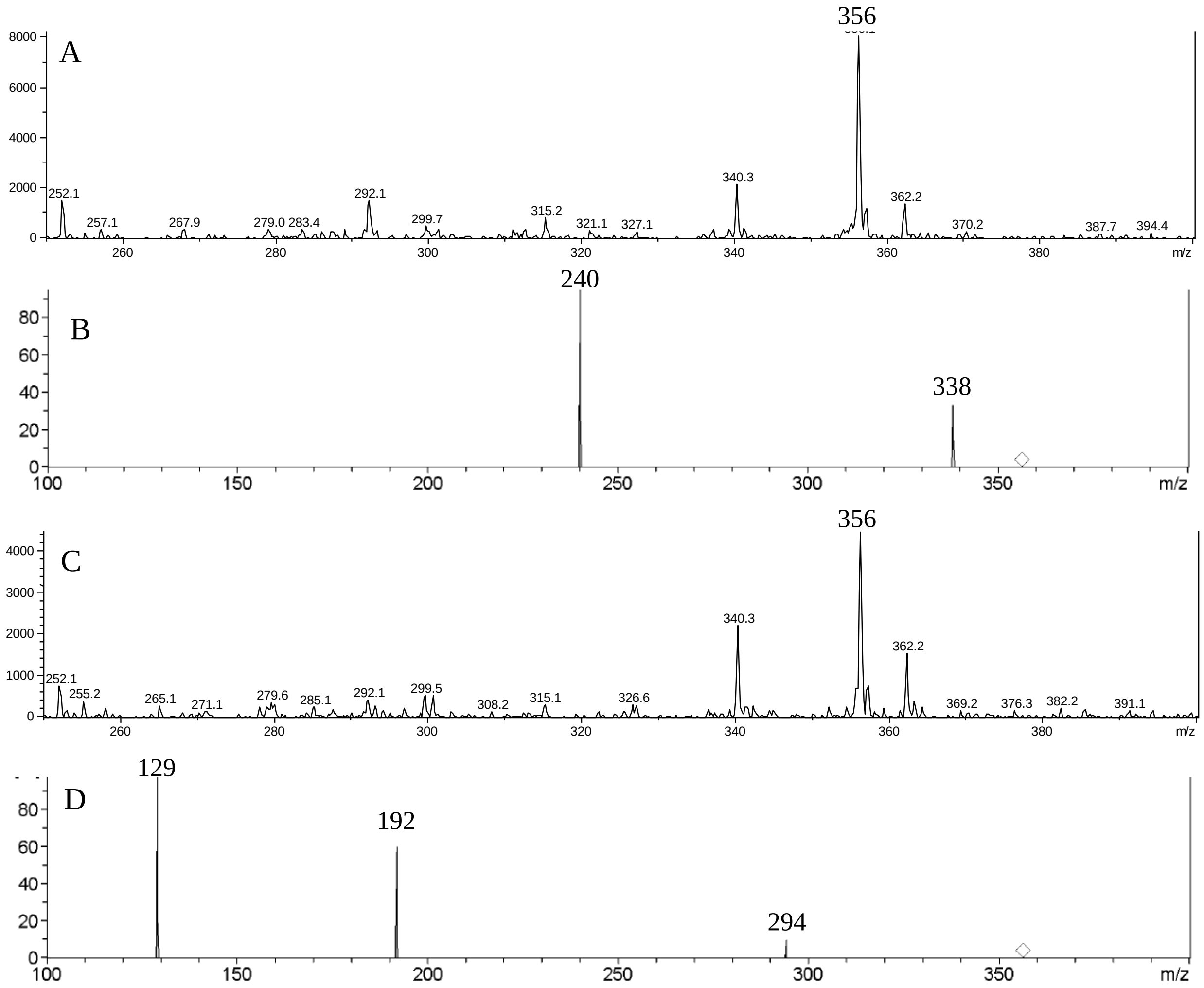

Figure-S8: MS/MS^n Spectra of **M8**: A) Mass spectrum of **M8a** (*m/z* = 356; RT = 31.3 min.); B) MS^2 spectrum of **M8a** (at *m/z* = 356); C) Mass spectrum of **M8b** (*m/z* = 356; RT = 31.7 min.); D) MS^2 spectrum of **M8b** (at *m/z* = 356)

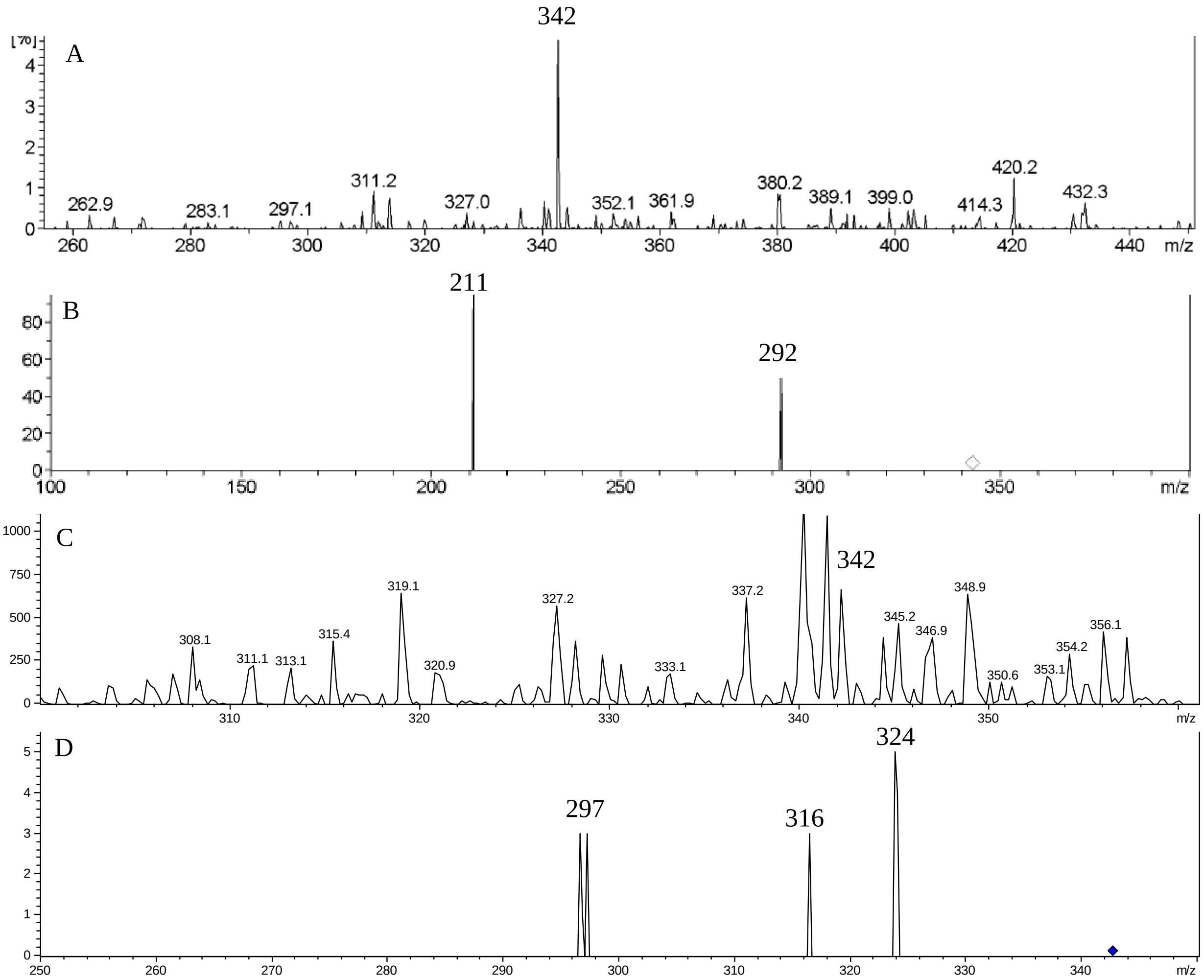

Figure-**S9a/b**: MS/MS^n Spectra of **M9a/b**: A) Mass spectrum of **M9a** (*m/z* = 342; RT = 25.9 min.); B) MS^2 spectrum of **M9a** (at *m/z* = 342); C) Mass spectrum of **M9b** (*m/z* = 342; RT = 26.4 min.); D) MS^2 spectrum of **M9b** (at *m/z* = 342)

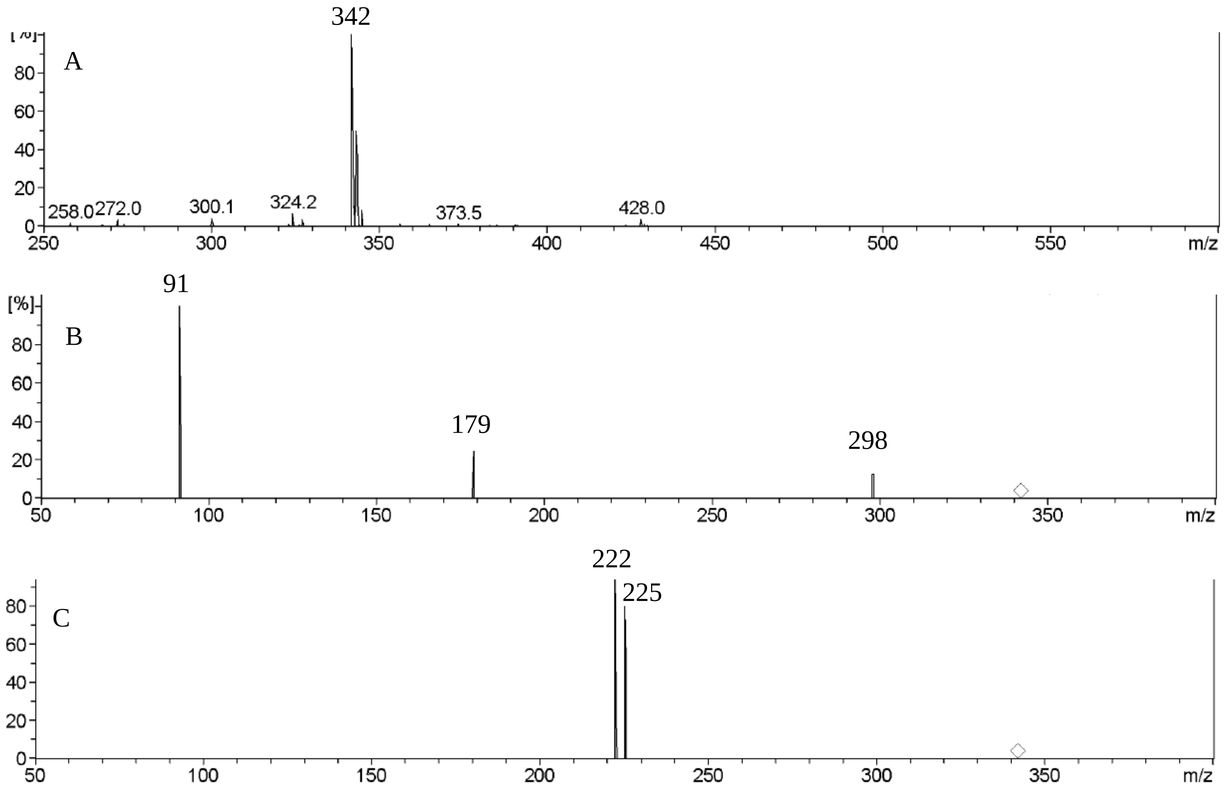

Figure-**S9c/d**: MS/MS^n Spectra of **M9c/d**: A) Mass spectrum of **M9c/d** (*m/z* = 342; RT = 6.1 or 6.4 min.); B) MS^2 spectrum of **M9c/d** (at *m/z* = 342; RT = 6.1 min.); C) MS^2 spectrum of **M9d/c** (at *m/z* = 342; RT = 6.4 min.)

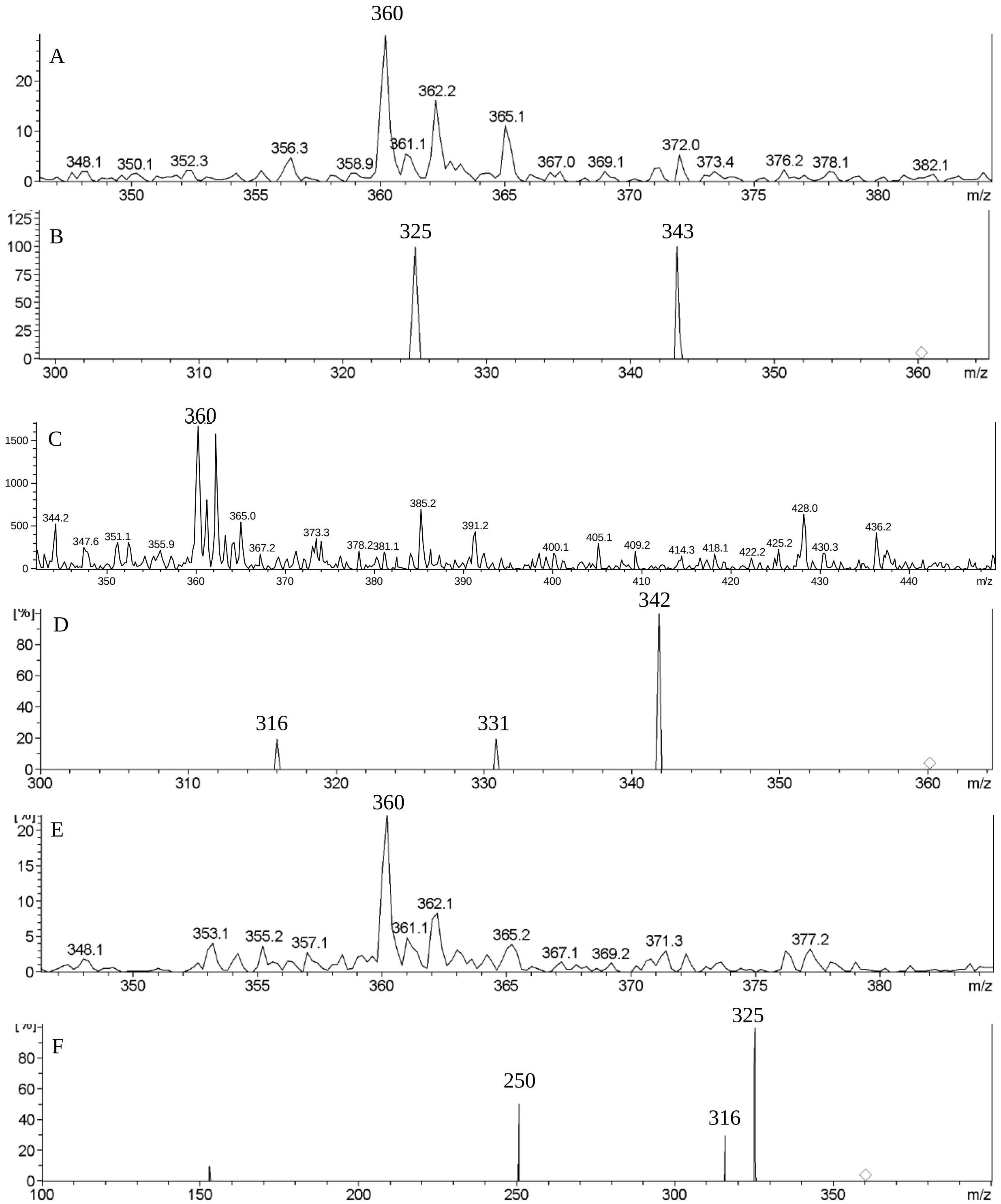

Figure-**S10**: MS/MS^n Spectra of **M10**: A) Mass spectrum of **M10a** (*m/z* = 360; RT = 20.8 min.); B) MS^2 spectrum of **M10a** (at *m/z* = 360); C) Mass spectrum of **M10b** (*m/z* = 360; RT = 22.8 min.); D) MS^2 spectrum of **M10b** (at *m/z* = 360); E) Mass spectrum of **M10c** (*m/z* = 360; RT = 23.8 min.); F) MS^2 spectrum of **M10c** (at *m/z* = 360)

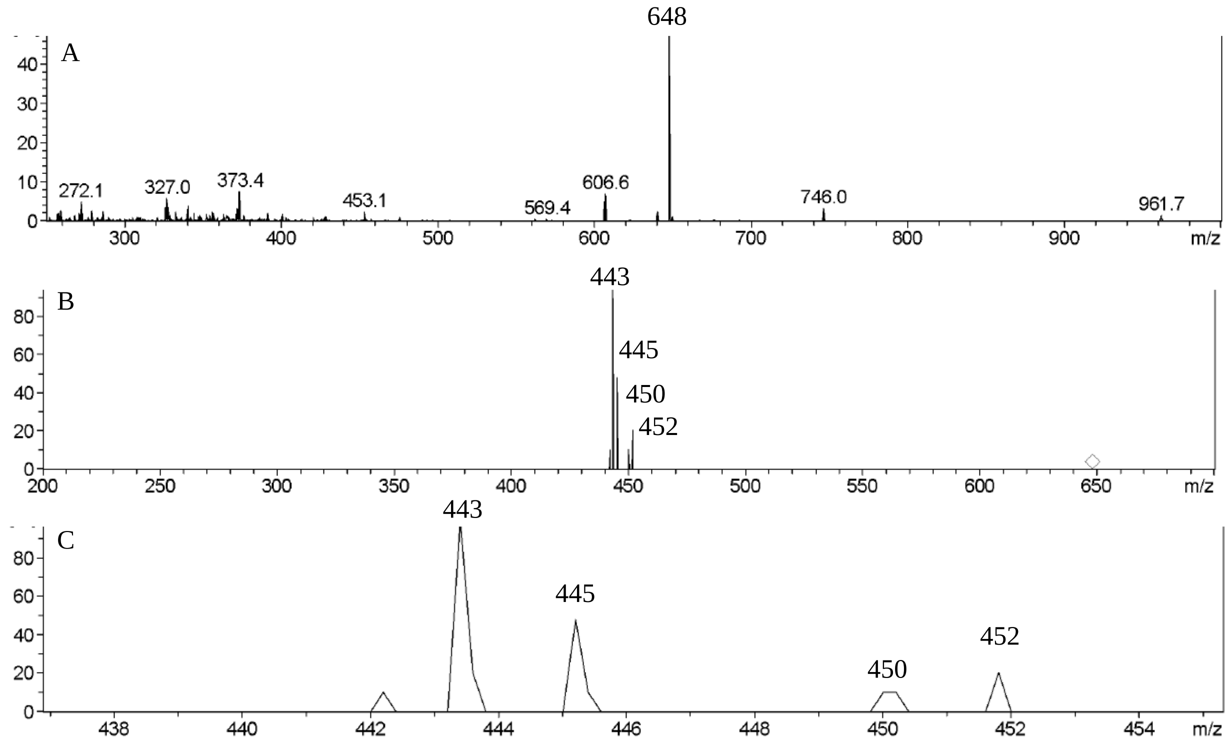

Figure-**S11**: MS/MS^n Spectra of **M11**: A) Mass spectrum of **M11** (m/z = 648; RT = 10.5 min.); B) MS^2 spectrum of **M11** (at m/z = 648); C) Expanded **11B**

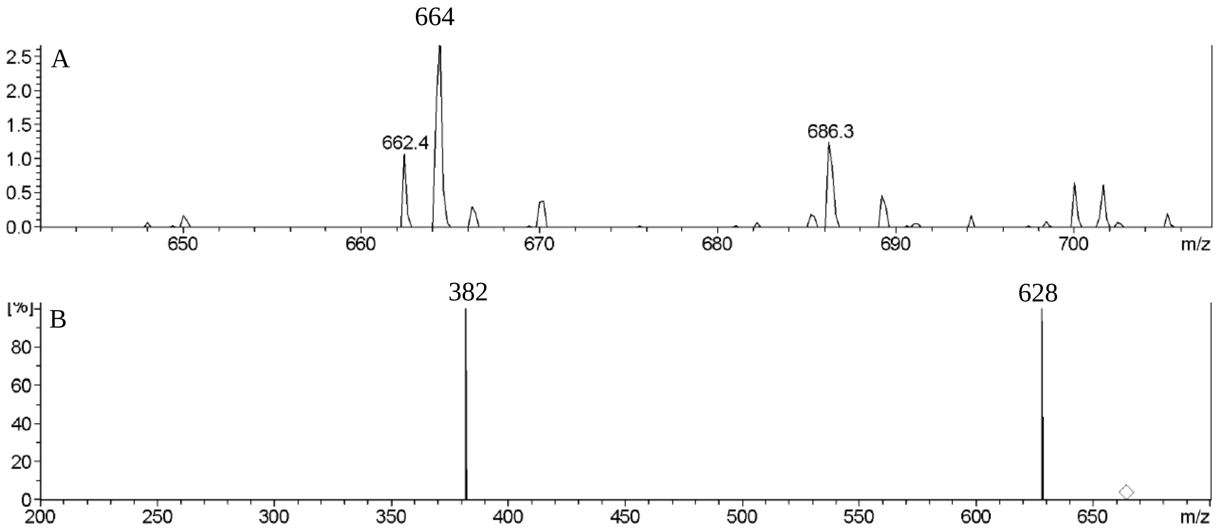

Figure-**S12**: MS/MS^n Spectra of **M12**: A) Mass spectrum of **M1** (m/z = 664; RT = 20.6 min.); B) MS^2 spectrum of **M12** (at m/z = 664)

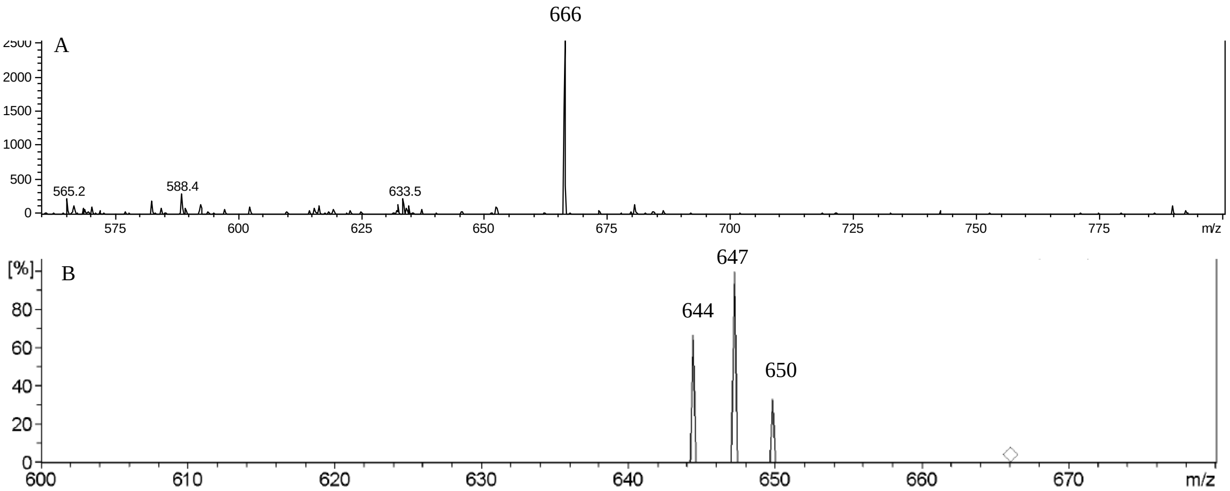

Figure-**S13**: MS/MS^n Spectra of **M13**: A) Mass spectrum of **M13** (*m/z* = 666; RT = 20.5 min.); B) MS^2 spectrum of **M13** (at *m/z* = 666)

Table-S1. RLMs incubations of Galeon

Incubation type	Microsomes	Conc.	NADPH	$MgCl_2$	GSH	$MeONH_2$	Buffer
Test 1	40µL	1µL	100µL	100µL			759µL
Test 2	40µL	1µL	100µL	100µL			759µL
Test 3	40µL	1µL	100µL	100µL	100µL		659µL
Test 4	40µL	1µL	100µL	100µL	100µL		659µL
Test 5	40µL	1µL	100µL	100µL		250µL	509µL
Test 6	40µL	1µL	100µL	100µL		250µL	509µL
Control 1	40µL	0	100µL	100µL			760µL
Control 2	40µL	1µL	0µL	100µL			859µL
Control 3	0 µL	1µL	100µL	100µL			799µL
Control 4	40µL	0	100µL	100µL	100µL		660µL
Control 5	40µL	1µL	0µL	100µL	100µL		759µL
Control 6	0 µL	1µL	100µL	100µL	100µL		699µL
Control 7	40µL	0	100µL	100µL		250µL	510µL
Control 8	40µL	1µL	0µL	100µL		250µL	609µL
Control 9	0 µL	1µL	100µL	100µL		250µL	549µL

Table-S2. LC Gradient solvent system

Time (min.)	Water (H_2O)	Acetonitrile (ACN)
0	95	5
5	95	5
10	70	30
20	40	60
30	10	90
40	40	60
50	70	30
60	95	5
